# Altered Extracellular Vesicle-Derived Protein and microRNA Signatures in Bronchoalveolar Lavage Fluid from Patients with Chronic Obstructive Pulmonary Disease

**DOI:** 10.3390/cells13110945

**Published:** 2024-05-30

**Authors:** Sabine Bartel, Justina C. Wolters, Hasnat Noor, Karim Rafie, Jiahua Fang, Benedikt Kirchner, Esther Nolte-′t Hoen, Michael W. Pfaffl, Steven Rutgers, Wim Timens, Maarten van den Berge, Machteld N. Hylkema

**Affiliations:** 1 Department of Pathology and Medical Biology, University of Groningen, University Medical Center Groningen, 9713 GZ Groningen, The Netherlands; 2 Groningen Research Institute for Asthma and COPD (GRIAC), University of Groningen, University Medical Center Groningen, 9713 GZ Groningen, The Netherlands; 3 Department of Pediatrics, University of Groningen, University Medical Center Groningen, 9713 GZ Groningen, The Netherlands; 4 Department of Molecular Pharmacology, Groningen Research Institute of Pharmacy (GRIP), University of Groningen, 9712 CP Groningen, The Netherlands; 5 Division of Animal Physiology and Immunology, School of Life Sciences Weihenstephan, Technical University of Munich, 85354 Freising, Germany; 6 Institute of Human Genetics, LMU University Hospital, LMU Munich, 80539 Munich, Germany; 7 Department of Biomolecular Health Sciences, Faculty of Veterinary Medicine, Utrecht University, 3584 CS Utrecht, The Netherlands; 8 Scheper Hospital, 7824 AA Emmen, The Netherlands; 9 Department of Pulmonary Diseases, University of Groningen, University Medical Center Groningen, 9713 GZ Groningen, The Netherlands

**Keywords:** chronic obstructive pulmonary disease (COPD), extracellular vesicle (EV), proteomics, miRNA, bronchoalveolar lavage (BAL), inflammation

## Abstract

Chronic obstructive pulmonary disease (COPD) is a progressive lung disease for which there is no cure. Accumulating research results suggest a role for extracellular vesicles (EVs) in the pathogenesis of COPD. This study aimed to uncover the involvement of EVs and their molecular cargo in the progression of COPD by identification of EV-associated protein and microRNA (miRNA) profiles. We isolated EVs from the bronchial alveolar lavage fluid (BALF) of 18 patients with COPD and 11 healthy controls using size-exclusion chromatography. EV isolates were characterized using nanoparticle tracking analysis and protein content. Proteomic analysis revealed a higher abundance of 284 proteins (log2FC > 1) and a lower abundance of 3 proteins (log2FC < −1) in EVs derived from patients with COPD. Ingenuity pathway analysis showed that proteins enriched in COPD-associated EVs trigger inflammatory responses, including neutrophil degranulation. Variances in surface receptors and ligands associated with COPD EVs suggest a preferential interaction with alveolar cells. Small RNAseq analysis identified a higher abundance of ten miRNAs and a lower abundance of one miRNA in EVs from COPD versus controls (Basemean > 100, FDR < 0.05). Our data indicate that the molecular composition of EVs in the BALF of patients with COPD is altered compared to healthy control EVs. Several components in COPD EVs were identified that may perpetuate inflammation and alveolar tissue destruction.

## 1. Introduction

COPD is characterized by persistent airway inflammation, destruction of the extracellular matrix in the lung (emphysema), and chronic mucus hypersecretion, all contributing to irreversible airway obstruction [1]. Current treatments provide symptomatic relief, but do not address the progression of emphysema. Understanding the molecular mechanisms behind these processes is crucial to develop effective therapies or preventative strategies. 

Recent research indicates the involvement of extracellular vesicles (EVs) in various lung diseases. In addition, circulating plasma-derived EVs have been shown to have great potential to be used for the diagnosis, prognosis, and therapeutics of lung diseases such as COPD [2]. EVs, released by virtually all cells in the body, are categorized based on the size and origin into exosomes (<150 nm) derived from multivesicular bodies in endosomes, and microvesicles (~100 nm → 1 μm) shed from the cell surface [3]. EVs functionally transfer various content, including miRNAs, between cells [4]. It is believed that the uptake of EVs is in part regulated by the receptor/ligand interaction with the target cells [5], but relevant receptor/ligand pairs for the interaction of EVs with distinct lung cells are not yet well understood. 

Substantial research has linked EVs and their cargo to lung disease (reviewed in [6]), but there are a lack of data regarding their role in COPD. EVs from activated human neutrophils containing neutrophil elastase have been shown to be present in lavage fluid of patients with COPD and to cause tissue destruction resembling emphysema when applied to mice [7]. In contrast, EVs released by mesenchymal stem/stromal cells aid tissue regeneration and dampen inflammation [8], underscoring the varied roles of different EV types in the development and progression of COPD.

Here, we aimed to decipher the distinct molecular signature of EVs in the lungs of patients with COPD. EV-enriched fractions from bronchial alveolar lavage fluid (BALF) were isolated from 11 healthy controls and 18 patients with COPD, and subsequently subjected to untargeted proteomics and small RNA sequencing. Ingenuity pathway analysis (IPA) and a hierarchical clustering analysis of protein abundance revealed that COPD EVs exhibit a different protein and miRNA content, which may enhance the inflammatory responses and degranulation of cells. Specifically, among other proteins, we identified a different presence of surface receptor and ligands on the COPD EVs that could indicate a different cellular origin and an enhanced targeting of alveolar cells of the EVs in COPD compared to controls.

## 2. Materials and Methods

### 2.1. Study Population

All study participants were included in a clinical study performed in the UMCG, as described earlier [9]. Briefly, the study included 11 healthy controls without a history of pulmonary disease and healthy lung function, and 18 patients with moderate COPD (GOLD stage II). All participants were free of asthma and atopy, and all but three non-smoking individuals were ex-smokers with a median of 26.5 pack years (Table 1). Patients were not on medication. Thirteen subjects who had received maintenance treatment with inhaled corticosteroids discontinued this one month prior to the study. Exclusion criteria were treatment with oral corticosteroids and antibiotics or a respiratory tract infection in the month prior to the study. The protocol was approved by the hospital ethics committee. All patients gave their informed consent [9].

### 2.2. Collection and Processing of Bronchoalveolar Lavage

The collection and processing of BALF has been described previously [9]. Briefly, BALF was collected during a bronchoscopy with a flexible fiberoptic bronchoscope (Olympus B1 IT10; Olympus Optical, Tokyo, Japan) in a subsegmental bronchus of the middle lobe. For lavage, four 50 mL aliquots of normal saline pre-warmed to 37 °C were inserted and immediately recovered using a negative pressure of <2.7 kPa (<20 mmHg). 

BALF was filtered through a 48 mm nylon gauze (Curapharm249.1 filter; Maxxim Medical Europe, ’s-Hertogenbosch, The Netherlands) and recovery and aspect were assessed. After centrifuging for 5 min at 400× *g* at 4 °C, supernatants were decanted and immediately stored in aliquots at −80 °C.

### 2.3. Isolation of EVs from BAL Fluid

**Figure 1:**
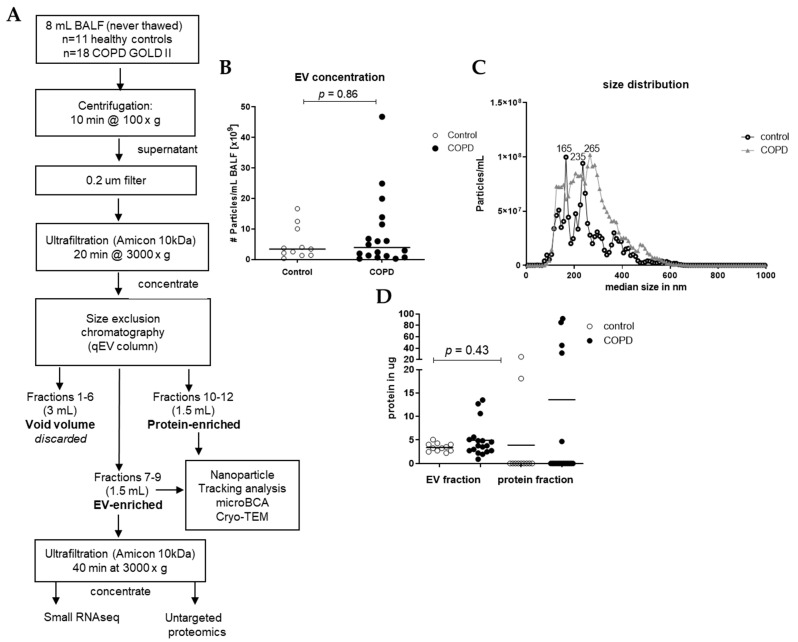
Isolation workflow and characterization of EVs from BALF of healthy controls (n = 11) and patients with COPD (n = 18). (**A**) Isolation workflow. Isolated EVs were characterized using nanoparticle tracking analysis for the (**B**) concentration and (**C**) size and (**D**) microBCA for protein content. Data are expressed as mean; Mann–Whitney U for COPD vs. control.

EVs were isolated from 8 mL of each sample using different steps of centrifugation, ultrafiltration, and size exclusion chromatography (SEC), as shown in Figure 1. In short, preliminary cellular debris and large vesicles were removed using centrifugation at 1500× *g* for 10 min (Multifuge X pro Thermo Scientific centrifuge, TX-1000 (7500317). The BALF was further concentrated and purified using an Amicon filter, filter type 10 kDa (Merck Milipore, Darmstadt, Germany), which was centrifuged at 3000× *g* for 20 min to concentrate the BALF to 0.5 mL, and this was added to a size SEC qEV original column (35 nm, serial #: 1001659, Izon, Christchurch, New Zealand). Subsequently, 5 × 0.5 mL Hank’s Balanced Salt Solution (HBSS, Lonza Bioscience, Walkersville, MD, USA) was added to the column. These 3 mL was referred to as void volume, which was discarded. Consequently, 6 times 0.5 mL HBSS was added to the column, and the first 3 fractions (1.5 mL) were collected in an Eppendorf (EV-enriched fraction) and the last 3 fractions (1.5 mL) were collected in an Eppendorf (protein-enriched fraction). Of the EV-enriched fraction, 1.4 mL was put in an Amicon filter and centrifuged at 3000× *g* for 40 min to concentrate and purify the EV fraction. After centrifugation, approximately 80 μL was collected and stored at −80 °C until untargeted proteomics, small RNAseq analysis, and Cryo-transmission electron microscopy (Cryo-TEM). The remaining of the EV-enriched fraction and the protein-enriched fraction of the samples were stored at −20 °C until microBCA and nanoparticle tracking analysis (NTA). To assess the concentration of EVs in the isolated SEC fractions, we measured all samples with NTA using a Nanosight camera device (version NTA 3.0 0060, Nanosight, Malvern, UK). The NTA is a powerful technique for characterizing nanoparticles based on their Brownian motion and light-scattering properties, providing valuable insights into the size distribution and concentration of nanoparticles in BALF. All samples were applied to the machine in 1 mL of PBS (1:100 dilution), and three videos of 1 min each were taken and combined for the analysis. The dilution was adjusted individually to achieve between 10–100 particles/frame during the measurement. 

### 2.4. Cryo-TEM Imaging

Cryo-TEM was employed to directly visualize vesicle integrity. Initially, a 50 μL EV-enriched sample from a patient with COPD was concentrated to 5 μL using a 500 μL Amicon Ultra Centrifugal filter with a 10 kDa MWCO (Merck, UFC5010BK). Subsequently, a 3 μL aliquot of the concentrated sample was deposited onto glow-discharged holey carbon-coated grids (3.5/1 Quantifoil Micro Tools, Jena, Germany). After the excess liquid was blotted, the grids were vitrified in liquid ethane utilizing a Vitrobot (FEI, Eindhoven, The Netherlands) and then transferred to a FEI Tecnai T20 electron microscope equipped with a Gatan model 626 cryo-stage operating at 200 keV. Micrographs were captured under low-dose conditions using a slow-scan CCD camera. Images are shown in Supplementary Figure S1.

### 2.5. Protein Concentration Measurement

Protein measurement was performed with a microBCA kit (ThermoFisher Scientific, Rockford, IL, USA) according to the manufacturer’s recommendations. Briefly, 5 μL of the isolated EV or protein fractions was diluted with 95 μL of PBS in a 96-well microplate and was incubated with BCA reagent for 1 h at 37 °C. Each plate contained a standard row of declining BSA concentrations in duplicates (200 μg/mL; 50 μg/mL; 25 μg/mL; 12.5 μg/mL, 6.25 μg/mL, 3.125 μg/mL; 1.56 μg/mL and 0 μg/mL). Absorbance at 562 nm was assessed using a plate reader (ClarioStart Plus, BMG Labtech, Ortenberg, Germany).

### 2.6. Untargeted Proteomics Analysis 

Protein levels were determined using discovery-based proteomics (using label-free quantification) for relative protein concentrations as described previously [10], but with alterations in the LC-MS detection method and subsequent data analyses. 

In short, in-gel digestion was performed on 30 μL isolated EV material containing an equivalent of the EV isolation from 4 mL of BALF of each sample. Tryptic digestion (1:100 *g*/*g* sequencing grade modified trypsin V5111; Promega, Madison, WI, USA) was performed after reduction with 10 mmol/L dithiothreitol and alkylation with 55 mmol/L iodoacetamide proteins before the peptides were eluted from the gel for mass spectrometric analyses. 

Chromatographic separation of the peptides was performed using liquid chromatography (LC) on a nano-HPLC system (Ultimate 3000, Dionex, Sunnyvale, CA, USA) using a nano-LC column (Acclaim PepMapC100 C18, 75 µm × 50 cm, 2 µm, 100 Å, Dionex, buffer A: 0.1% *v*/*v* formic acid, dissolved in milliQ-H2O, buffer B: 0.1%, *v*/*v* formic acid, dissolved in acetonitrile). In general, an equivalent of 60% of the digested starting material from the isolated EVs was injected using the µL-pickup method with buffer A as a transport liquid from a cooled autosampler (5 °C) and loaded onto a trap column (µPrecolumn cartridge, Acclaim PepMap100 C18, 5 µm, 100 Å, 300 µm × 5 mm, Dionex). Peptides were separated on the nano-LC column using a linear gradient from 2–45% buffer B in 117 min at a flowrate of 300 nL/min. The mass spectrometer was operated in positive ion mode and data-independent acquisition mode (DIA) using isolation windows of 12 *m*/*z* with a precursor mass range of 300–1200. LC-MS raw data were processed with Spectronaut (version 15.1.210713.50606) (Biognosys, Schlieren, Switzerland) using the standard settings of the directDIA workflow, with a human SwissProt database (www.uniprot.org, 20.350 entries, accessed 15 November 2019). 

### 2.7. RNA Isolation

After SEC isolation, half of the concentrated EV fraction (30 μL) was directly added to 700 μL of Qiazol (Qiagen, Venlo, The Netherlands). Isolation of small RNAs was performed with the miRNeasy micro kit (Qiagen) according to the manufacturer’s recommendations. RNA was eluted in 14 μL water and stored at −80 °C until the entire sample was subjected to small RNA sequencing.

### 2.8. Small RNA Sequencing

Sequencing of small RNAs, including miRNAs, was performed as previously described using the NEBNext Multiplex Small RNA Library Prep Set for Illumina (New England Biolabs, Ipswich, MA, USA) on a HiSeq 2500 (Illumina, San Diego, CA, USA) with minor modifications to the manufacturer’s protocol [11]. In brief, all samples were reduced to a minimal volume using a vacuum centrifuge and were resuspended in 8 µL water. In total, 6 µL thereof was used for NGS library preparation using the NEBNext Multiplex Small RNA Library Prep Set for Illumina (New England Biolabs, Ipswich, MA, USA) with minor modifications to the manufacturer’s protocol. Adaptors were ligated to RNA, reverse transcription was performed, and the product was barcoded and amplified using PCR. The size distribution and quantity of resulting cDNA was assessed using a DNA 1000 Kit on a 2100 Bioanalyzer (Agilent Technologies, Santa Clara, CA, USA) before size selection with high-resolution agarose gel electrophoresis. The size range of miRNA-containing cDNA fragments was cut from the gel and purified. After verifying the final library by capillary gel electrophoresis (DNA High Sensitivity Kit, Agilent Technologies), 50 cycles of single-end sequencing were performed on a HiSeq 2500 (Illumina, San Diego, CA, USA).

### 2.9. Statistical Analysis

#### 2.9.1. Proteomics Analysis 

For the quantification, the Q-value filtering was set to the classic setting without imputing, which included unpaired *t*-testing for the calculation of the differential abundances between the groups. Raw data with FDR values were exported from the Spectronaut software (version 15.1.210713.50606), and for downstream processing, we only included proteins that were detected in >70% of all samples (20/29). Although a disparity in smoking status between the two groups, due to the small sample size and the predominance of ex-smokers, we did not control for smoking. However, we conducted a correlation analysis on the Foldchange of significant proteins, comparing all samples, with and without the three non-smoking COPD patients. This analysis demonstrated a high degree of correlation, indicating that the inclusion of non-smoking patients did not significantly impact our findings (see Supplementary Figure S2).

#### 2.9.2. Ingenuity Pathway Analysis

Ingenuity pathway analysis (IPA, Qiagen) was performed on proteins and/or miRNAs with a log2FC < −1 or >1 and false discovery rate (FDR) < 0.05) in EVs from COPD patients vs. controls. To this end, we used the IPA function “core analysis”. Selected candidate pathways were chosen according to the level of activation (z-score), significance (*p* < 0.05, Fisher’s exact *t*-test), and biological relevance to the research question.

#### 2.9.3. Protein Clustering and Functional Annotation 

Two-way hierarchical clustering was employed on the top 50 proteins, focusing on their divergence in abundance from control subjects, but independent of both the False Discovery Rate (FDR) and log2 fold change (log2FC). This method categorized proteins into different groups based on their expression patterns, facilitated by ranking proteins based on their standard deviation, ensuring robustness in the selection process, resulting in the creation of condensed heatmaps. The ComplexHeatmap R/Bioconductor package (Gu 2016) was employed to create heatmaps using scaled log-expression values (z-score), applying Euclidean distance and Ward linkage. The standard deviation for ranking and subsequent functional annotation of protein clusters was carried out using the bigOmics analytics tool [12].

To explore the potential involvement in immune pathways, the correlation between protein clusters and functional annotations from immune-related reference sets, available in the Gene Ontology (GO) databases, was examined. The Fisher test for weighting was used to determine the robustness of these correlations.

#### 2.9.4. Correlations of Protein Abundance with Macrophage and Neutrophil Cell Number in BALF

Protein abundance from each of the four clusters were correlated with the number of macrophages and neutrophils per 1 mL of BALF of patients with COPD and controls, as well as the lung function parameter FEV1/FVC using Spearman’s test. The quantification of macrophages and neutrophils in BALF was performed using the methods previously described in reference [8]. 

#### 2.9.5. microRNA Expression Profiling

Raw sequencing data were trimmed of tailing adapter sequences with Btrim [13] prior to discarding reads with less than 16 nt in length. To avoid false positive findings, reads mapping to human rRNA, tRNA, snRNA, or snoRNA sequences [14] were filtered from the data set, and remaining reads were directly aligned to mature miRNA sequences obtained from iRbase (v21, [15]) using a Bowtie short read aligner [16]. Differential expression analysis, including the normalization and multiple testing correction using the Benjamini–Hochberg approach, was performed using the DESeq2 package [17] in R [18].

All statistical comparisons (besides the proteomics or miRNAseq analysis) between the two groups were conducted using a Mann–Whitney U test with a threshold of significance of *p* < 0.05. All analysis was performed in GraphPad Prism 8. 

## 3. Results

### 3.1. Small EVs Can Successfully Be Isolated from Frozen BALF Samples

Small EVs were isolated from 8 mL of never-thawed BALF aliquots using size exclusion chromatography (SEC) (Figure 1A). Nanoparticle tracking analysis (NTA) of the EV-enriched SEC fractions revealed a median particle count of 3.42 × 10^9^ particles/mL for healthy controls and 3.92 × 10^9^ particles/mL for COPD samples (Figure 1B). The median size of isolated particles was similar for controls (248 nm) and for COPD BALF (249 nm) (Figure 1C). Protein quantities were sufficient to proceed to proteomics (Figure 1D). EV-containing fractions were concentrated using ultrafiltration, and equal volumes were subjected to both RNA isolations for small RNA-sequencing and an untargeted proteomics analysis (Figure 1A). Cryo-TEM images of isolated EVs showed that EVs were intact and had a round shape (Figure S1).

### 3.2. Proteomics Analysis of BALF EVs

**Figure 2:**
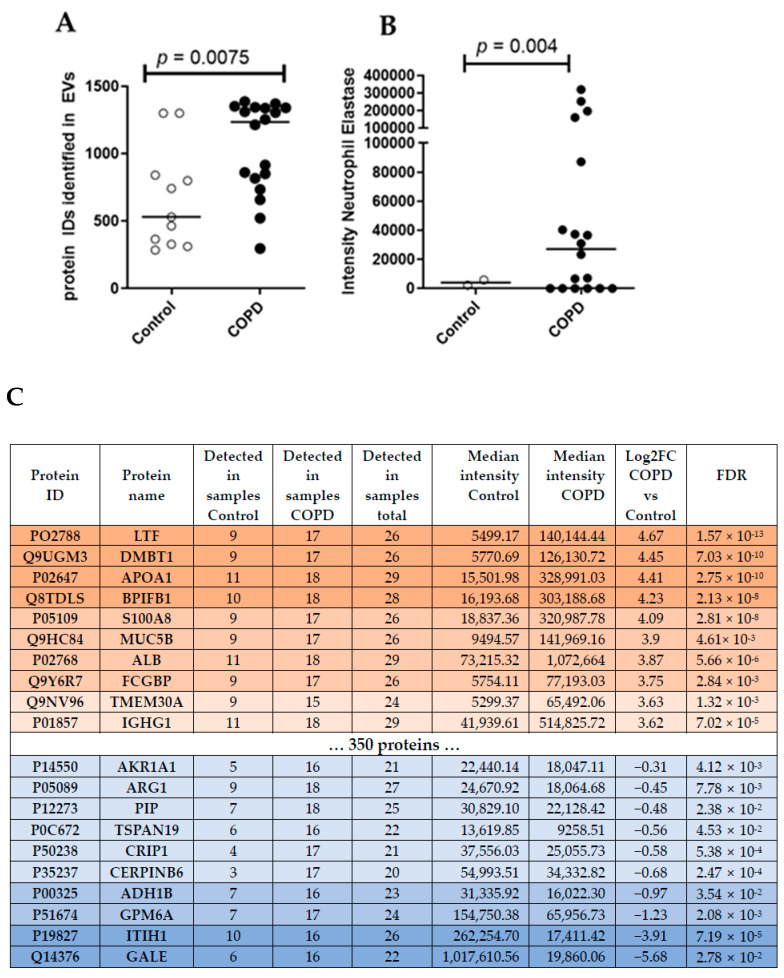
(**A**) Number of unique peptides identified using LC/MS untargeted proteomics analysis. Mann–Whitney U vs. control. (**B**) Intensity of Neutrophil Elastase in BALF EVs. (**C**) Top ten differentially abundant proteins in COPD subjects.

Unbiased proteomics analysis using LC/MS identified more unique peptides in BALF EVs derived from patients with COPD (median = 1234) compared to healthy controls (median = 531) (Figure 2A). According to the guidelines for minimal information essential for studies of EVs (MISEV) [19], several protein markers need to be present and/or absent from the isolated EVs to judge their integrity and purity. Table 2 lists all markers of the MISEV2018 guidelines and their detection intensity in our samples. Accordingly, we were able to detect the majority of proteins that are frequently found to be present in EVs (i.e., category 1 and 2, Table 2), suggesting that we indeed enriched EVs from our BALF samples. We also detected a contamination with lipoproteins (category 3a, Table 2), as expected by a purely SEC-based isolation [20]. Interestingly, the intensity of most EV-specific markers (Table 2) was higher in COPD samples, indicating a clear enrichment of BALF EVs in COPD.

To compare different EV-associated protein signatures between COPD and control samples, we focused on the 596 proteins that were detected in at least 70% of all samples (20/29). This approach identified 370 proteins that were significantly (FDR < 0.05) altered in COPD-derived BALF EVs. Thereby, three proteins were down-regulated in COPD EVs compared to controls (log2FC < −1) and 284 proteins were up-regulated (log2FC > 1) (Top10 listed in Figure 2C, see Table S1 in the online supplement for the complete list). The top three up-regulated proteins in COPD BALF EVs were Lactotransferrin (LTF, log2FC = 4.67, FDR = 1.57 × 10^−13^), Deleted In Malignant Brain Tumors 1 (DMBT1, log2FC = 4.45, FDR = 7.03 × 10^−6^), and Apolipoprotein A1 (APOA1, log2FC = 4.41, FDR = 2.75 × 10^−10^). UDP-Glucose-4-Epimerase (GALE, log2FC = −5.68, FDR = 2.78 × 10^−2^), Inter-Alpha-Trypsin Inhibitor Heavy Chain 1 (ITIH1, log2FC = −3.91, FDR = 7.19 × 10^−5^), and Glycoprotein M6A (GPM6A, log2FC = −1.23, FDR = 2.08 × 10^−3^) were the top three down-regulated proteins in COPD BALF EVs. We also detected the previously described protein Neutrophil Elastase, important in emphysema development, in all samples of COPD patients, but only in few controls (Figure 2B).

**Figure 3:**
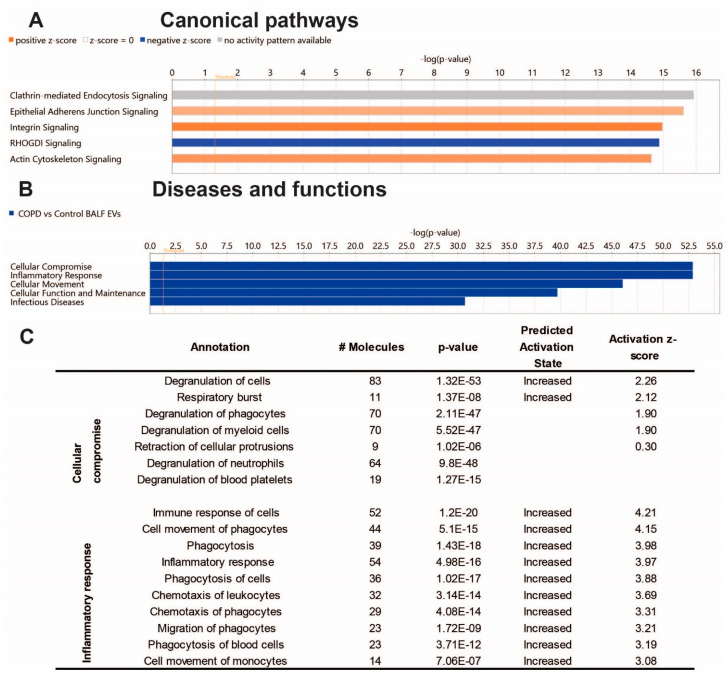
Ingenuity pathway analysis of the BALF EV proteome Core analysis using Ingenuity Pathway Analysis (Qiagen). (**A**) Subsection ‘Canonical Pathways’ (cut-off −log10, *p*-value > 14); (**B**) subsection ‘Diseases & Functions’ (cut-off −log10, *p*-value > 30), and the Table showing associated annotations of the top two functions ‘cellular compromise’ and ‘inflammatory response’ with activity predictions. (**C**) Image derived from ‘regulator effects’ prediction.

To gain insight into the biological effects of the altered protein content of COPD EVs, we performed Ingenuity Pathway Analysis (IPA). As shown in Figure 3A, IPA analysis revealed proteins associated with COPD-derived BALF EVs most significantly involved in the canonical pathways clathrin-mediated endocytosis signaling, epithelial adherens junction signaling, and integrin signaling. The latter two were predicted to be higher in COPD EVs, while Rho GDP-dissociation inhibitor (RHOGDI) signaling was predicted to be lower in COPD EVs compared to healthy controls. The IPA annotation ‘diseases and functions’ showed that the protein content of COPD BALF EVs is associated with cellular compromise, inflammatory response, and cellular movement (Figure 3B). Subsections of these annotations are listed in Figure 3C and show that IPA predicts the activation of, for example, the degranulation and immune response of cells, indicating that EVs in the lung might be functionally involved in COPD pathogenesis. 

**Figure 4:**
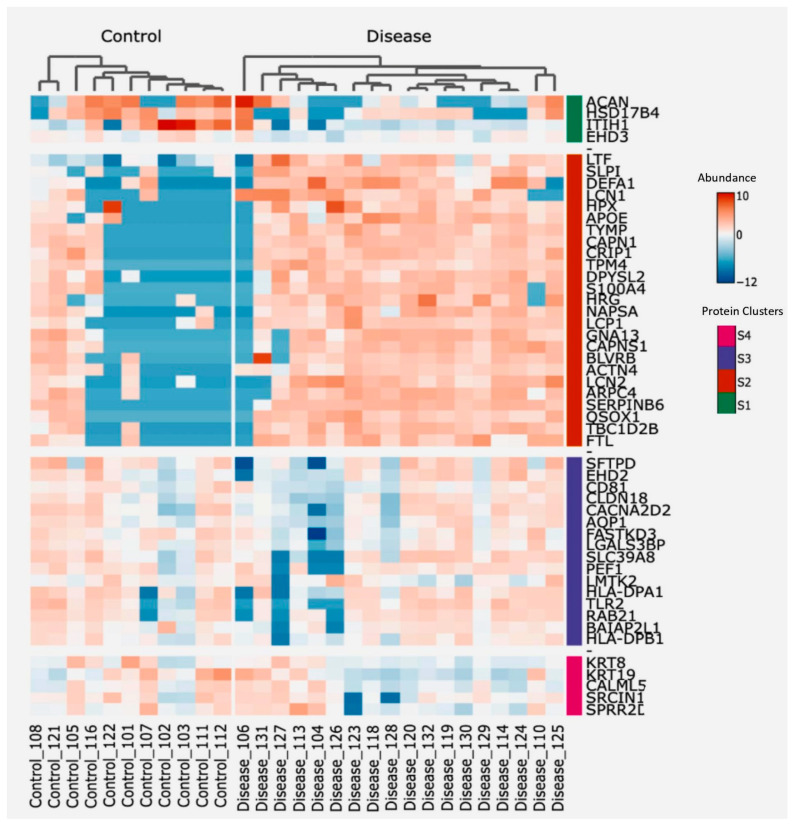
Heatmap showing protein abundance of the top 50 proteins, sorted using two-way hierarchical clustering. Red corresponds to the higher and blue corresponds to the lower abundance of the protein. Based on abundance, protein groups are divided into four clusters; S1 (Green), S2 (Orange), S3 (Purple), and S4 (Pink).

To identify proteins potentially associated with the destructive-immune response in COPD, we conducted a two-way hierarchical clustering analysis focusing on the top 50 proteins ranked by a standard deviation metric. This analysis generated a clustered heatmap displaying protein abundance, with annotated protein groups categorized into four distinct clusters based on their functionalities (Figure 4). Next, we cross-referenced the significantly differentially abundant proteins from our dataset with immune-related datasets to identify correlations (Table 3), showing that the second and third cluster proteins contain the main clusters with a positive correlation to immune-related responses, whereas cluster 4 proteins were negatively correlated to one of the immune-related pathways. Additionally, proteins from each of the four clusters were correlated with both the count of macrophages and neutrophils per mL of BALF from the identical samples used for EV isolation, as well as the lung function parameter FEV1/FVC. 

The results presented in Table 4 reveal significant correlations between the abundance of specific proteins with macrophage and neutrophil numbers in BALF. In controls, both lectin galactoside-binding soluble 3-binding protein (LGALS3BP) and BAIAP2L1 encoding insulin-responsive protein of mass 53 kD (IRSp53) correlated negatively with BALF macrophage numbers (r = −0.60, *p* = 0.03 and r = −0.67, *p* = 0.02, respectively). Conversely, in COPD EVs, Apolipoprotein E (APOE) exhibited a negative correlation with macrophage numbers (r = −0.49, *p* = 0.04), while Aquaporin1 (AQP1) showed a positive correlation (r = 0.51, *p* = 0.03) with macrophage numbers. Notably, the abundance of proteins from these four clusters did not display significant correlations with neutrophil numbers in BALF from the same individuals. 

In relation to lung function, noteworthy correlations emerged between specific proteins in EVs and the FEV1/FVC parameter. In control EVs, abundance of Lipocalin-2 (LCN2) correlated positively to FEV1/FVC (r = 0.60, *p* = 0.03), whereas abundance of Apolipoprotein E (APOE) and the neutrophil granule protein Lactoferrin (LTF) correlated negatively to FEV1/FVC (r = −0.45, *p* = 0.05 and r = −0.50, *p* = 0.04, respectively) in COPD EVs.

### 3.3. Altered Receptor/Ligand Pattern on BALF EVs in COPD

**Figure 5:**
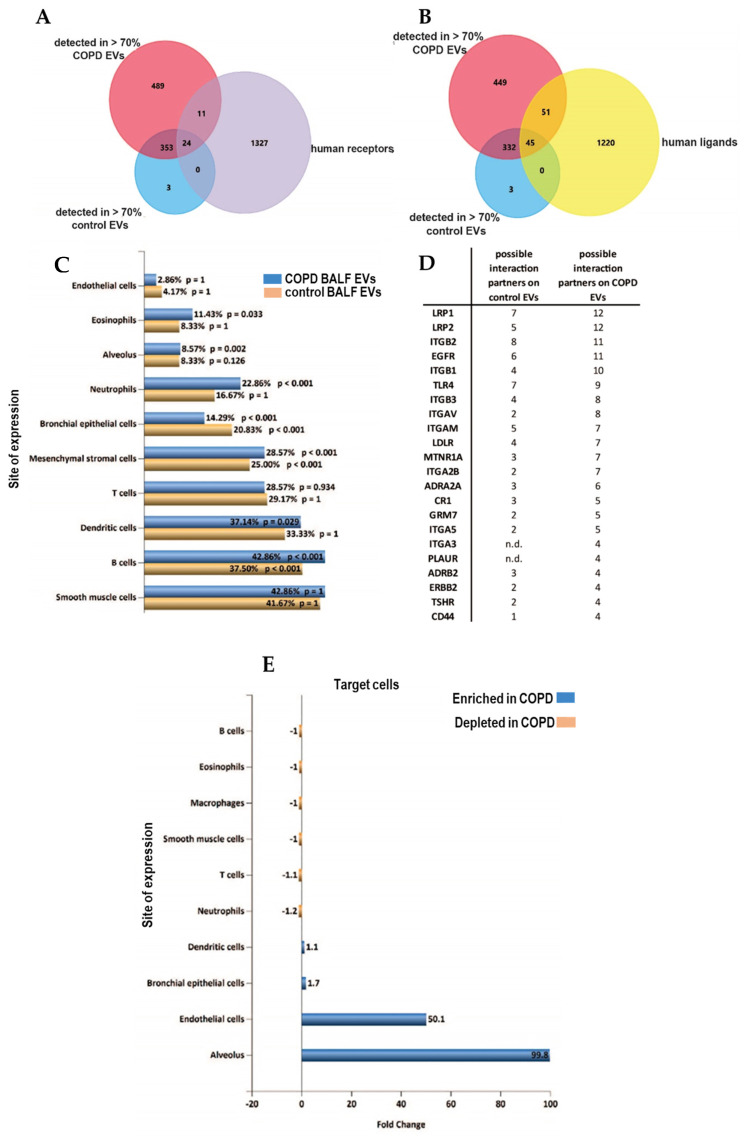
Altered receptor/ligand patterns on the EV surface of BALF EVs in COPD. (**A**) Venn Diagrams to determine the expression of receptors (purple) and (**B**) ligands (yellow) derived from CellTalkDB (yellow) on EVs from both COPD (red) or healthy control EVs (blue). (**C**) Cellular origin of EVs predicted using the detected receptors on all BALF EVs (yellow), and receptors detected on COPD EVs (blue) using FUNRICH ‘site of expression’ analysis. (**D**) List of cellular receptors and amount of ligands on BALF EVs that are possible interaction partners. (**E**) FUNRICH site of expression analysis of the cellular receptors addressed by control or COPD BALF EVs.

Identification of receptors on EVs can potentially be used to delineate the cells of EV origin, while characterization of ligands may identify target cell types with which EVs can interact. We screened the proteins detected in >70% of samples of each group for all identified human receptors and ligands (derived from CellTalkDB [21]). Of the 35 identified receptors, 11 were found on COPD EVs but not in controls (Figure 5A, Table S2 of online supplement). Our BALF EV dataset contained 96 ligands, of which 51 ligands were enriched in COPD EVs (Figure 5B, Table S3 in online supplement). By using the EV analysis tool Funrich [22], we were able to associate the receptors on our BALF EVs to their cellular origin. Figure 5C shows that BALF EVs derived from COPD patients seem to be derived relatively more from immune cells, such as neutrophils, eosinophils, or macrophages. To identify the potential target cells, we listed the respective receptors that are paired to the ligands on the BALF EVs (Figure 5D) and the number of ligands in our dataset that are able to interact with these receptors according to CellTalkDB [21]. Accordingly, the receptors for which ligands are present on control BALF EVs from healthy individuals are Integrin beta 2 (ITGB2, 8 interactions), LDL receptor-related protein 1 (LRP1, 7 interactions), and Toll-like receptor 4 (TLR4, 7 interactions). COPD BALF EVs are enriched in ligands that can interact with LRP1 (12 interactions), LRP2 (12 interactions), and epidermal growth factor receptor (EGFR, 11 interactions) (Figure 5D). 

A Funrich site of expression analysis revealed an almost 100-fold enrichment of interactions between the EVs in COPD and the alveolus (Figure 5E), and interactions with endothelial cells were predicted to be enriched 50-fold.

### 3.4. miRNAseq Analysis of BALF EVs

**Figure 6:**
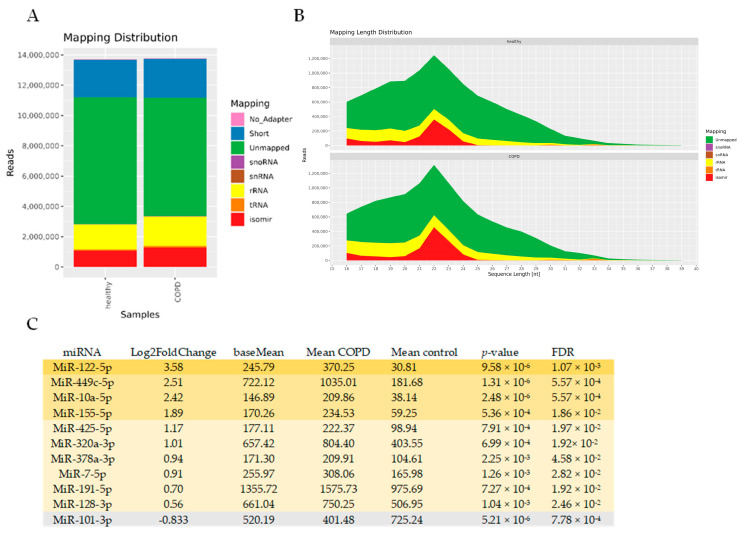
Altered miRNA expression in BALF-derived EVs of COPD patients compared to healthy controls. (**A**) Mapping distribution of small RNA-sequencing expressed in read counts (**B**) and length. (**C**) Table with ten up- and one down-regulated miRNAs with a base mean of >100 and FDR < 0.05.

To identify miRNAs associated with EVs in COPD, we performed an unbiased RNA-seq analysis of isolated BALF EVs. In general, sequencing was successful in all 29 samples, with high quality scores. The mapping distribution revealed a relatively small yet expected percentage of miRNAs in the sequencing reads (~10%), which still reached 1 million reads (Figure 6A,B) and a large percentage of unmapped reads (which might include truncated RNAs). Of the 1400 detected miRNAs, 96 miRNAs had a base mean expression value of >100, ensuring sufficient expression across both COPD and control EVs (see Supplementary Table S4 for the full list). Eleven miRNAs showed differential abundance between COPD and controls with an FDR < 0.05 (Figure 6C). Ten miRNAs showed higher abundance (miR-122-5p, miR-449c-5p, miR-10a-5p, miR-155-5p, miR-425-5p, miR-320a-3p, miR-378a-3p, miR-7-5p, miR-191-5p, and miR-128-3p), whereas one miRNA was detected in lower abundance (miR-101-3p) compared to healthy controls (Figure 6C). Due to the low number of differentially abundant miRNAs, we did not perform an IPA analysis. Abundance of miRNAs was also not correlated with immune cell numbers and lung function. 

## 4. Discussion

This is the first study to investigate both the protein and small RNA content of EVs derived from BALF samples of patients with COPD and healthy controls. We identified 284 up- and 3 down-regulated proteins in COPD BALF EVs compared to healthy controls, and 10 up-regulated and one down-regulated miRNA. Our data suggest that the protein signature found in pulmonary EVs in COPD sustains inflammatory reactions and cellular dysfunction. Analyzing the receptor and ligand patterns on the EVs provided suggestive evidence that EVs in COPD originated from immune cells rather than structural cells, and that their potential target cells reside in the alveolar region. The suggestion of immune cell-derived COPD EVs was supported by the correlations between protein abundance and macrophage numbers in the corresponding BALF samples. Consequently, the altered composition in EVs in the BALF reflects the predominant feature of COPD, which is persistent inflammation, resulting potentially in an imbalance between tissue damage and repair in the alveolar target cells.

The most prominently up-regulated protein in COPD EVs was Lactotransferrin. LTF is known as an antimicrobial peptide released from goblet cells in the airway epithelium or from glands [23]. While LTF is a component of the airway lining fluid, it has also been reported to be present in BALF and increased in chronic bronchitis [23,24]. Although there has not been any prior association reported between LTF and EVs in the lung, recent discoveries have highlighted its presence, along with other antimicrobial peptides, in EVs secreted by human mesenchymal stem/stromal cells [25]. Additionally, studies have indicated its increased presence in EVs released from primary human neutrophils following in vitro stimulation [7]. Notably, in our study, we did not find a correlation with LTF abundance and the number of neutrophils in BALF. This suggests that LTF could be secreted by different cell types in COPD.

DMBT1, found to be up-regulated in COPD BALF EVs, belongs to the category of innate defense proteins. This protein has been described to be increased in an alveolar cell line following hypoxic conditions, where it plays a role in surfactant function [26]. Moreover, it has demonstrated the ability to stimulate repair mechanisms in alveolar cells by reducing IL-6 levels while enhancing vascular endothelial growth factor (VEGF) production [27]. Beyond its intracellular function, DMBT1 has been identified in human stem cell-derived EVs, which exhibit similar angiogenic properties and facilitate a repair-inducing effect [28]. These collective findings suggest that DMBT1 might contribute to repair and angiogenic mechanisms in the lung, potentially influencing repair mechanisms and surfactant modulation in the context of COPD.

The third most prominently up-regulated EV-associated protein in COPD, ApoA1, raises some intriguing possibilities. It has been considered to be a potential contaminant from lipoproteins during EV isolation, but may also be associated with EVs [29]. The observed four-fold increase in COPD samples compared to healthy controls may suggest a higher amount of lipoproteins in the BALF of COPD patients or a higher capacity of COPD EVs to bind lipoproteins. Studies in sputum from patients with mild COPD revealed lower ApoA1 expression compared to smokers without lung pathology [30]. In contrast, overexpression of ApoA1 in the alveolar epithelium protected mice from developing cigarette-smoke-induced emphysema [31]. These conflicting findings highlight the uncertainty surrounding the precise role of ApoA1 in the pathogenesis of COPD. 

The most down-regulated protein observed in COPD BALF EVs was GPM6A. Interestingly, prior reports have highlighted its secretion in EVs, a process influenced by stressors in the blood [32]. Additionally, GPM6A is possibly involved in Alzheimer’s disease [33]. According to the human protein atlas [34], GPM6A is primarily expressed in the alveolar epithelium. This particular location makes this a compelling protein to investigate in the context of COPD. Despite its secretion in EVs under stress conditions, and its association with Alzheimer’s disease, there is currently no documented report specifically linking GPM6A to human lungs or pulmonary EVs.

The top up-regulated EV-associated miRNAs-miR-122-5p, miR-449c-5p, miR-10a-5p and miR-155-5p-reveal intriguing connections within the context of COPD. Starting with miR-122-5p, it has been found to be downregulated in lung tissue-derived EVs of COPD patients in comparison to healthy smokers [35]. Further studies demonstrated its downregulation in sputum and plasma of COPD patients during exacerbation, indicating its role as a negative regulator of IL-17A production [36]. In plasma-derived EVs from patients with asthma, miR-122-5p was increased [10]. These contrasting effects in different compartments and diseases suggest that miRNA expression might depend on specific cell type activation within a particular context. Regarding the up-regulation of miR-449c-5p, our results align with a study in COPD demonstrating a higher expression of this miRNA in BAL-derived extracellular miRNAs of COPD patients compared to smokers and non-smokers [37]. Similarly, miR-10a-5p was found to be higher in bronchial epithelial cells from patients with asthma and COPD compared to controls [38]. Studies on miR-155-5p showed higher expression in the lung tissue of smokers without airflow limitation and patients with COPD compared to never-smokers, and its inhibition in mice demonstrated an attenuated CS-induced pulmonary inflammation, suggesting a role in COPD pathogenesis [39]. In our study, we observed increased miR-155 in BALF EV of COPD patients. This heightened expression, combined with the inflammatory proteins associated with EVs, could potentially contribute to the perpetuation of pulmonary inflammation in COPD.

We found some interesting correlations within the presence of specific proteins in BALF. In controls, both LGALS3BP and BAIAP2L1 correlated negatively with BALF macrophage numbers. LGALS3BP is a ubiquitously expressed cellular secreted glycoprotein with multiple antiviral activities. Its expression is stimulated by IFNs, resulting in upregulated 90 K serum concentrations in individuals with viral infections [40]. The negative association with macrophage numbers in controls could relate to the absence of infections in these healthy individuals. 

In COPD EVs, AQP1 showed a positive correlation with macrophage numbers. AQP1 functions as a water channel, fostering migration in various cell types [41]. However, in macrophages, the impact of AQP1-induced migration depends on external stimuli. In a model of acute bacterial peritonitis, mice lacking AQP1 (Aqp1−/−) displayed reduced infiltrating M1 macrophages when exposed to LPS stimulation [42]. Additionally, resting Aqp1−/− macrophages shifted from M0 to an M2 phenotype. This means that the observed correlation of AQP1 with macrophage numbers in COPD might signify a higher presence of activated M1 macrophages. 

With regard to lung function, specific proteins within EVs showed correlations with the FEV1/FVC parameter. In control EVs, abundance of Lipocalin-2 (LCN2) correlated positively to FEV1/FVC (r = 0.60, *p* = 0.03). This holds significance as LCN2 is known for its vital roles in airway defense, particularly in attenuating inflammation and aiding pathogen clearance [43]. Conversely, in COPD EVs, more LTF correlated with lower FEV1/FVC (r= −0.50, *p* = 0.04). This aligns with a recent study in a mouse model of particulate matter (PM)-induced pulmonary injury, where more LTF protein was found in PM-exposed lung tissues, indicating LTF’s possible involvement in lung injury triggered by PM exposure [44].

We realize that there are some limitations of our current study. The use of samples stored at −80 degrees Celsius for over 20 years [9] might have impacted sample quality. Although, since all samples were consistently handled and never thawed before this study, we are confident that these valuable samples can still be used to generate hypotheses regarding the role that EVs in the COPD lung pathology. Furthermore, we successfully identified a substantial number of proteins, including several classical EV markers, and achieved a sufficient sequencing depth of 1 million reads for miRNAs. The integrity of the EVs following the extended storage duration was confirmed through cryo-TEM. Regarding EV isolations containing lipoproteins, this occurrence was expected due to the method employed, specifically SEC, which does not effectively separate lipoproteins from small EVs [20]. Given the limited material and the complexity of employing multiple isolation techniques, complete separation would have been technically unfeasible [45]. Recognizing the presence of lipoproteins alongside EVs is important for future interpretations. 

Additionally, the utilization of in silico tools like IPA analysis and FunRich software (Version 3.1.4) provides an initial framework for insight and hypothesis generation based on established associations in the literature. However, it is important to acknowledge that these findings would benefit from confirmation through mechanistic experiments in future studies to validate and enhance their significance. Our cluster analysis confirmed the abundant presence of proteins linked to immune activation pathways. Furthermore, the correlations observed between the abundance of some of these proteins with macrophage numbers and lung function represent compelling points of interest. These correlations suggest potential links between specific proteins, immune response pathways, and clinical parameters, offering valuable insights into the complex interplay within COPD pathophysiology.

Our proteomics analysis identified more unique protein IDs in the COPD BALF EVs as compared to the control EVs. This disparity could arise from either a higher protein content of COPD EVs, or an increased quantity of EVs in BALF samples of patients with COPD. Although the NTA did not directly measure the EV count, the elevated expression intensity of classical EV markers, as defined by the MISEV2018 guidelines [19], might support the latter hypothesis. However, quantifying EVs using common markers such as tetraspanins might not be accurate, as their expression can significantly vary based on EV source and external stimuli [46]. Furthermore, evidence suggests that EVs can carry a protein corona on their surface, consisting of proteins like ApoA1, ApoB, ApoE, complement factors 3 and 4B, fibrinogen α-chain, immunoglobulin heavy constant γ2 and γ4 chains, many of which are found to be increased in our dataset [29]. Interestingly, studies demonstrated that EVs bearing a corona induce a stronger inflammatory response in human monocyte-derived dendritic cells compared to EV-free protein aggregates [29]. It is thus very intriguing to speculate that EVs in the BALF also carry such a protein corona, which might be functionally involved in COPD pathogenesis. 

In our study, there was a disparity in smoking status between the two groups. Due to the small sample size and the predominance of ex-smokers, we did not control for smoking. However, we conducted a correlation analysis on significant protein abundances comparing all samples, with and without the three non-smoking COPD patients. As depicted in Figure S2, this analysis demonstrated a high degree of correlation, indicating that the inclusion of non-smoking patients did not significantly impact our findings. Moreover, it is plausible that these non-smoking patients may have been exposed to passive or even third-hand smoke from a smoking partner or at the workplace. This can no longer be verified. 

## 5. Summary and Conclusions

This is the first study to investigate both the protein and small RNA content of EVs derived from BALF samples from patients with COPD and healthy controls. We show here that good quality EVs can be isolated from BALF samples that have been stored at −80 °C for an extended period of time. We also showed that the protein content of EVs is significantly altered in COPD and has an association with increased inflammatory responses and cellular damage in the lungs. BALF creates a unique opportunity to study the effects of EVs on the pathogenesis of COPD, given that the lower complexity of the biological fluid in this compartment and the identified targets of this study may already provide a good starting point for further targeting. Furthermore, vesicles are received directly from the disease microenvironment and may appear earlier in BALF than in peripheral circulation. More research is needed to decipher the complex role of EV-associated molecules in COPD pathogenesis, which could potentially also reveal novel therapeutic candidates.

## Figures and Tables

**Table 1:** Clinical characteristics.

	COPD (*n* = 18)	Controls (*n*-11)
Age (years)	62 ± 8	58 ± 8
Female/Male	4/14	3/8
Smoking status–ex-smokers (%)	84	100
Pack years	28 ± 22	25 ± 15
FEV_1_ (% pred) *	59 ± 13	104 ± 11
FEV_1_/FVC *	66 ± 11	97 ± 8
Bronchoalveolar lavage wash	18	11
Concentration of cells in lavage fluid		
Total cell count per mL	0.061 (0.01–0.6)	0.10 (0.03–0.2)
Macrophages (%)	86 (13.3–94.2)	92 (70.4–96.5)
Neutrophils (%)	1.7 (0.3–85.5)	1.1 (0.1–3.0)
Lymphocytes (%)	6.9 (1.3–26.4)	7.1 (2.5–26.5)
Eosinophils (%)	0.4 (0–1.7)	0.2 (0–0.45)

Data are presented as median (interquartile range), n, n (%), or mean ± sd, unless otherwise stated. FEV1: forced expiratory volume in 1 s; FVC: forced vital capacity. The Mann–Whitney U-test was performed, unless otherwise stated. *: *p* < 0.05 versus COPD. Table derived from [9].

**Table 2:** Assessment of MISEV EV markers.

MISEV Category	Protein	Detected in Control Samples (Median Intensity)	Detected in COPD Samples (Median Intensity)
***1—transmembrane or GPI-anchored proteins***			
**1a—non-tissue specific**	**CD63**	11/11 (15,509.93)	18/18 (92,003.89)
	**CD81**	11/11 (94,397.15)	18/18 (126,942.64)
	**CD82**	10/11 (13,925.97)	18/18 (45,828.48)
	**CD47**	8/11 (30,198.34)	17/18 (128,907.36)
	**GNA**	0/11	0/18
	**HLA-A**	9/11 (23,105.60)	15/18 (66,913.24)
	**H2-K/D/Q**	0/11	0/18
	**ITGA3**	3/11 (16,334.62)	15/18 (17,816.25)
	**ITGB2**	5/11 (28,874.69)	16/18 (43,729.40)
	**TFR2**	0/11	0/18
	**LAMP1**	3/11 (31,599.47)	15/18 (28,388.38)
	**SDC**	0/11	0/18
	**BSG**	0/11	0/18
	**ADAM10**	4/11 (24,082.33)	14/18 (47,027.64)
	**CD73**	0/11	0/18
	**CD55**	11/11 (29,733.75)	18/18 (162,760.10)
	**CD59**	11/11 (109,809.55)	18/18 (363,903.25)
**1b—tissue specific**	**CD9**	11/11 (185,367.57)	18/18 (612,304.21)
	**EPCAM**	5/11 (9394.25)	11/18 (9267.61)
	**TSPAN8**	8/11 (13,506.14)	17/18 (27,948.92)
	**CD14**	3/11 (18,100.96)	14/18 (20,410.54)
***2—cytosolic proteins recovered in EVs***			
**2a—lipid or membrane-binding ability**	**ESCRT-I/II/II**	0/11	0/18
	**TSG101**	11/11 (30,495.01)	18/18 (68,322.18)
	**ALIX (PDCD6IP)**	11/11 (26,746.72)	18/18 (134,175.34)
	**ARRDC1**	8/11 (22,419.05)	17/18 (114,076.60)
	**FLOT1**	7/11 (10,344.42)	16/18 (35,016.16)
	**CAV**	0/11	0/18
	**ANXA1**	11/11 (49,761.63)	18/18 (406,865.34)
	**HSC70**	0/11	0/18
	**HSP84**	0/11	0/18
	**ARF6**	2/11 (45,850.05)	13/18 (38,333.01)
	**SDCBP**	11/11 (49,047.33)	18/18 (322,430.85)
	**MAPT**	0/11	0/18
**2b—promiscuous incorporation into EVs**	**HSP70**	0/11	0/18
	**ACT**	0/11	0/18
	**TUB**	0/11	0/18
	**GAPDH**	11/11 (26,518.46)	18/18 (87,466.69)
***3—major components of non-EV co-isolated structures***			
**3a—lipoproteins**	**APOA1**	11/11 (15,501.97)	18/18 (328,991.03)
	**APOB**	8/11 (81,382.46)	17/18 (19,999.87)
	**APOB100**	0/11	0/18
	**ALB**	11/11 (73,215.32)	18/18 (1,072,664.00)
**3b—protein–protein/nucleic acid aggregates**	**UMOD**	0/11	0/18
***4—Transmembrane, lipid-bound soluble proteins associated with other intracellular compartments than PM/endosomes***			
**4a—nucleus**	**HIST1HC**	5/11 (16,701.19)	13/18 (27,412.26)
	**LMNA**	4/11 (9030.91)	14/18 (21,075.30)
**4b—mitochondria**	**IMMT**	0/11	0/18
	**CYC1**	0/11	0/18
	**TOMM20**	0/11	0/18
**4c—secretory pathway (endoplasmatic riticulum, Golgi apparatus)**	**CANX**	4/11 (1663.54)	6/18 (16,269.11)
	**HSP90B1**	3/11 (3891.60)	8/18 (8363.01)
	**BIP**	0/11	0/18
	**GM130**	0/11	0/18
**4d—autophagosome, cytoskeleton**	**ATG9A**	0/11	0/18
	**ACTN1**	6/11 (14,611.46)	17/18 (42,763.41)
	**KRT18**	0/11	0/18
***5—Secreted proteins recovered with EVs***			
**5a—cytokines and growth factors**	**TGFB1/2**	0/11	0/18
	**IFNG**	0/11	0/18
	**VEGFA**	0/11	0/18
	**FGF1/2**	0/11	0/18
	**PDGF**	0/11	0/18
	**EGF**	0/11	0/18
	**IL**	0/11	0/18
**5b—adhesion and extracellular matrix proteins**	**FN1**	4/11 (27,088.45)	16/18 (19,794.65)
	**COL12A1**	0/11	9/18 (12,138.44)
	**MFGE8**	0/11	0/18
	**LGAL3BP**	0/11	0/18
	**CD5L**	6/11 (10,115.46)	16/18 (16,718.95)
	**AHSG**	5/11 (17,509.83)	16/18 (27,092.54)

**Table 3:** Correlation of the four clusters identified in Figure 4 with immune-related pathways based on a Fisher testing correlation analysis.

Annotations	S1	S2	S3	S4
GO_POSITIVE_REGULATION_OF_IMMUNE_SYSTEM_PROCESS	0	0.44	0.36	0
GO_REGULATION_OF_IMMUNE_RESPONSE	0	0.41	0.37	0
Innate Immune System_Homo sapiens_R-HSA-168249	0	0.34	0.17	0
GO_INNATE_IMMUNE_RESPONSE	0	0.53	0.31	0
GO_POSITIVE_REGULATION_OF_IMMUNE_RESPONSE	0	0.43	0.32	0
GO_IMMUNE_EFFECTOR_PROCESS	0	0.51	0	0
neutrophil activation involved in immune response (GO_000228)	0	0.53	0.62	−0.27
GO_ACTIVATION_OF_IMMUNE_RESPONSE	0	0.23	0.36	0
GO_REGULATION_OF_IMMUNE_EFFECTOR_PROCESS	0	0.30	0	0
GO_NEGATIVE_REGULATION_OF_IMMUNE_SYSTEM_PROCESS	0	0.34	0.34	0
GO_REGULATION_OF_INNATE_IMMUNE_RESPONSE	0	0.48	0.24	0
GO_ADAPTIVE_IMMUNE_RESPONSE	0	0	0	0
GO_POSITIVE_REGULATION_OF_INNATE_IMMUNE_RESPONSE	0	0.43	0.29	0
Hepatitis, Autoimmune C0241910 mouse GSE867 sample 230 (down)	0	0.56	0	0
GO_ACTIVATION_OF_INNATE_IMMUNE_RESPONSE	0	0	0.35	0
GO_HUMORAL_IMMUNE_RESPONSE	0	0.67	0	0
innate immune response activating cell surface receptor signaling pathway.	0	0	0	0

**Table 4:** Correlation of protein groups with FEV1/FVC and number of macrophages and neutrophils in BALF.

Parameter	Protein	Correlation	*p*-Value
**Lung function**			
Controls	LCN2	0.60	0.03
COPD	APOE	−0.45	0.05
	LTF	−0.50	0.04
**Macrophages in BALF**			
Controls	LGALS3BP	−0.60	0.03
	BAIAP2L1	−0.67	0.02
COPD	APOE	−0.49	0.04
	AQP1	0.51	0.03
**Neutrophils in BALF**			
Controls	-	-	-
COPD	-	-	-

## Data Availability

The mass spectrometry proteomics data have been deposited to the ProteomeXchange Consortium via the PRIDE [47] partner repository with the dataset identifier PXD049998.

## Supplementary Materials

The following supporting information can be downloaded at: https://www.mdpi.com/article/10.3390/cells13110945/s1, Figure S1: Images of COPD-EVs using cryo-TEM. Figure S2: Correlation analysis on significant protein abundances comparing all samples with and without non-smoking COPD patients. Table S1: Complete list of detected proteins in >70% of all samples; Table S2: Receptors on BALF EVs; Table S3: Ligands on BALF EVs; Table S4: List of detected miRNAs in BALF EVs.
