# Supplementary material for: Altered Extracellular Vesicle-Derived Protein and microRNA Signatures in Bronchoalveolar Lavage Fluid from Patients with Chronic Obstructive Pulmonary Disease
Source: Cells. 2024 May 30;13(11):945. doi: 10.3390/cells13110945 (PMC11171984; doi:10.3390/cells13110945)
Supplement: Supplementary file 1 [file cells-13-00945-s001.zip › cells-2824770-supplementary.pdf]

Altered Extracellular Vesicle-derived protein and microRNA signatures in bronchoalveolar lavage fluid from patients with Chronic Obstructive Pulmonary Disease

Sabine Bartel[1,2], Justina C. Wolters[3], Hasnat Noor[1,2], Karim Rafie[4], Jiahua Fang[1,2], Benedikt Kirchner[5,6], Esther Nolte-'t Hoen[7], Michael W. Pfaffl[5], Steven Rutgers[8], Wim Timens[1,2], Maarten van den Berge[2,9], Machteld N. Hylkema[1,2]*

* Correspondence can be sent to m.n.hylkema@umcg.nl

Affiliations

1. Department of Pathology and Medical Biology, University of Groningen, University Medical Center Groningen, University of Groningen, 9713 GZ Groningen , The Netherlands.
2. Groningen Research Institute for Asthma and COPD (GRIAC), University of Groningen, University Medical Center Groningen, 9713 GZ Groningen, The Netherlands.
3. Department of Pediatrics, University of Groningen, University Medical Center Groningen, 9713 GZ Groningen, The Netherlands.
4. Department of Molecular Pharmacology, Groningen Research Institute of Pharmacy (GRIP), University of Groningen, 9712 CP Groningen, The Netherlands
5. Division of Animal Physiology and Immunology, School of Life Sciences Weihenstephan, Technical University of Munich, 85354 Freising, Germany.
6. Institute of Human Genetics, LMU University Hospital, LMU Munich, 80539 Munich, Germany.
7. Department of Biomolecular Health Sciences, Faculty of Veterinary Medicine, Utrecht University, 3584 CS Utrecht, The Netherlands.
8. Department of Pulmonary Diseases, University Medical Center Groningen, University of Groningen, 9713 GZ Groningen, The Netherlands.

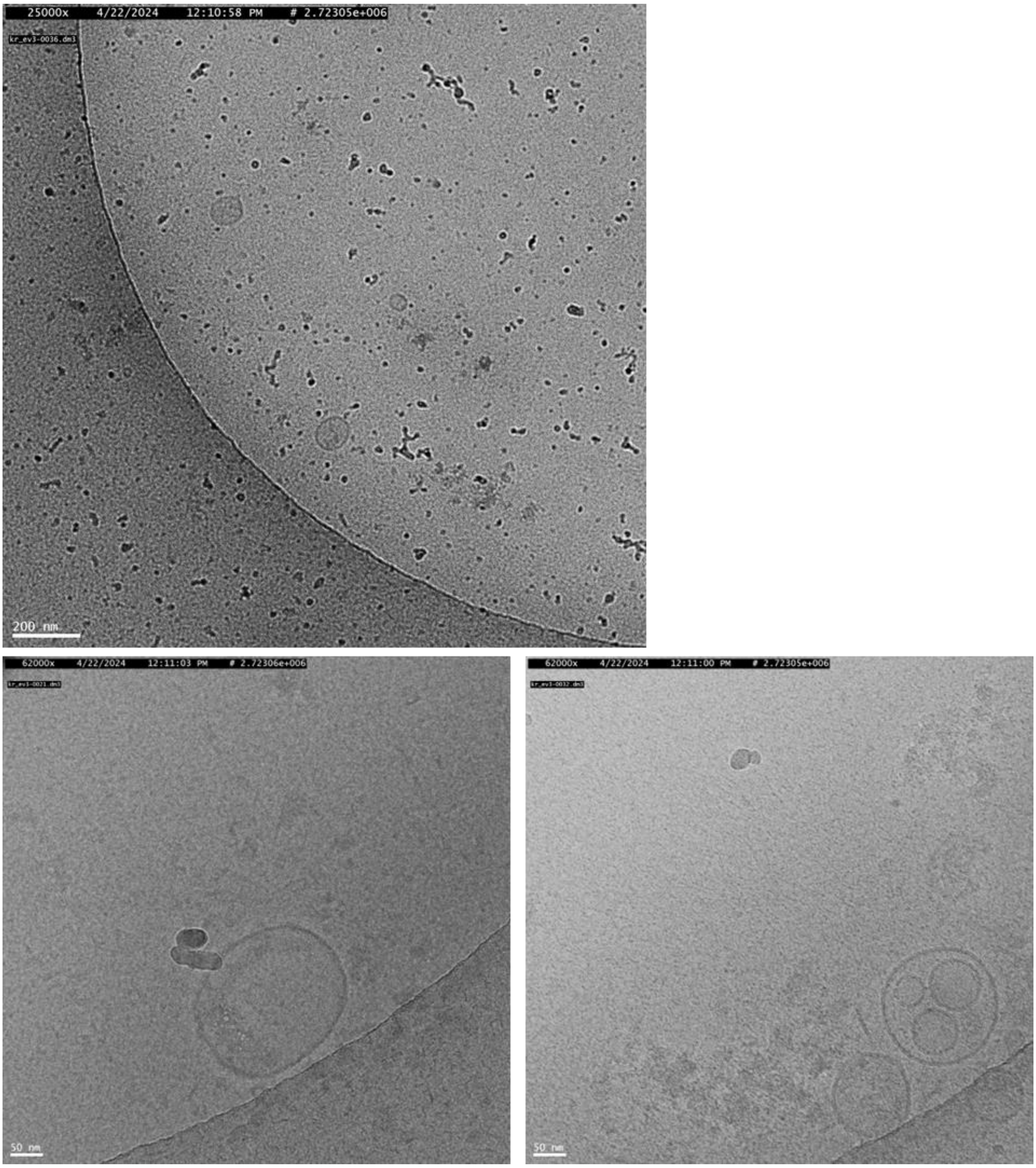

Figure S1. Images of COPD-EVs using cryo-transmission electron microscopy (Cryo-TEM)

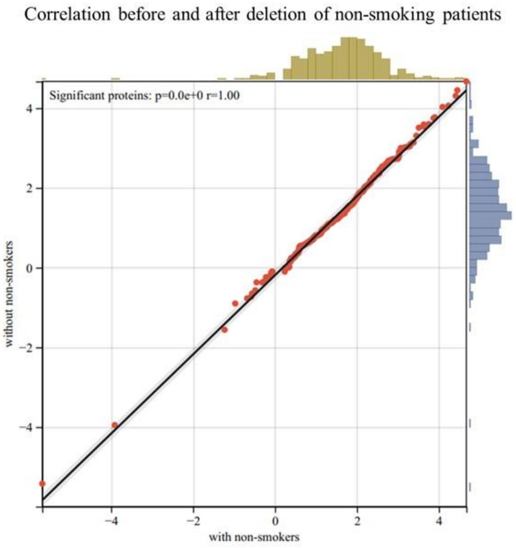

Figure S2. Correlation analysis on significant protein abundances comparing all samples with and without 3 non-smoking COPD patients

Table S1. Complete list of detected proteins in > 70% of all samples.

protein ID	protein name	detected in control	detected in COPD	median control	median COPD	log2FC COPD	FDR
**P02788**	**LTF**	9	17	5499.17	140144.44	4.67	1.57E-13
**Q9UGM3**	**DMBT1**	9	17	5770.69	126130.72	4.45	7.03E-06
**P02647**	**APOA1**	11	18	15501.98	328991.03	4.41	2.75E-10
**Q8TDL5**	**BPIFB1**	10	18	16193.68	303188.68	4.23	2.13E-08
**P05109**	**S100A8**	9	17	18837.36	320987.78	4.09	2.81E-08
**Q9HC84**	**MUC5B**	9	17	9494.57	141969.16	3.90	4.61E-03
**P02768**	**ALB**	11	18	73215.32	1072664.00	3.87	5.66E-06
**Q9Y6R7**	**FCGBP**	9	17	5754.11	77193.03	3.75	2.84E-03
**Q9NV96**	**TMEM30A**	9	15	5299.37	65492.06	3.63	1.32E-03
**P01857**	**IGHG1**	11	18	41939.61	514825.72	3.62	7.02E-05
**P01871**	**IGHM**	8	17	22371.98	254433.41	3.51	1.50E-04
**P98088**	**MUC5AC**	9	17	7163.80	78424.59	3.45	2.42E-09
**P01024**	**C3**	11	18	12868.19	134053.30	3.38	1.23E-04

**P01624**	**IGKV3-15**	9	17	22646.06	224035.34	3.31	9.51E-06
**P08133**	**ANXA6**	11	18	12007.96	117858.40	3.29	5.01E-09
**P28068**	**HLA-DMB**	6	15	6976.51	67504.19	3.27	2.38E-05
**P06702**	**S100A9**	8	16	11734.85	109878.65	3.23	1.55E-11
**Q96FQ6**	**S100A16**	7	15	2418.19	22125.20	3.19	1.58E-04
**Q9C0K1**	**SLC39A8**	11	15	12723.75	112727.95	3.15	4.98E-04
**P25815**	**S100P**	7	17	8317.23	69973.36	3.07	4.88E-04
**P08238**	**HSP90AB1**	11	18	12646.91	106302.48	3.07	4.15E-08
**P04083**	**ANXA1**	11	18	49761.63	406865.34	3.03	5.11E-10
**P22392**	**NME2**	5	15	6460.12	52598.04	3.03	7.23E-07
**P05026**	**ATP1B1**	5	15	8182.16	66202.91	3.02	6.46E-06
**P07988**	**SFTPB**	7	17	19427.78	156619.09	3.01	3.24E-05
**A0A0B4J1V0**	**IGHV3-15**	3	17	10186.30	81770.22	3.00	3.78E-09
**P01877**	**IGHA2**	11	18	73257.54	561263.61	2.94	5.44E-05
**O14493**	**CLDN4**	9	16	10389.07	76339.52	2.88	7.88E-03
**P0C0L4**	**C4A**	10	16	10357.59	74361.35	2.84	1.51E-03
**O00159**	**MYO1C**	11	18	16812.00	117246.56	2.80	3.44E-03
**P07195**	**LDHB**	7	18	6716.75	46171.30	2.78	8.83E-03
**P01591**	**JCHAIN**	9	17	32413.76	220953.47	2.77	7.95E-05
**P07355**	**ANXA2**	11	18	73958.42	502609.55	2.76	5.18E-07
**P80188**	**LCN2**	5	16	13513.51	91605.94	2.76	3.58E-12
**A0A075B6K4**	**IGLV3-10**	6	16	7809.60	51904.69	2.73	5.69E-07
**P31946**	**YWHAB**	11	18	12519.56	82877.36	2.73	6.08E-05
**Q71U36**	**TUBA1A**	8	16	25401.80	167786.43	2.72	8.65E-07
**O00560**	**SDCBP**	11	18	49047.34	322430.86	2.72	2.40E-03
**P01701**	**IGLV1-51**	5	16	11299.12	74205.03	2.72	8.06E-08
**P01833**	**PIGR**	10	18	25752.37	168829.40	2.71	1.77E-05
**P15328**	**FOLR1**	10	18	21321.14	138366.46	2.70	8.27E-03
**Q71DI3**	**HIST2H3A**	9	17	20775.53	133447.80	2.68	6.33E-07
**P14618**	**PKM**	10	18	12794.73	80711.59	2.66	3.08E-06
**P13639**	**EEF2**	11	18	11091.44	68115.48	2.62	3.67E-06
**P62937**	**PPIA**	11	18	26856.43	163805.85	2.61	2.68E-04
**P02675**	**FGB**	8	16	8114.62	49456.70	2.61	1.35E-05
**P02652**	**APOA2**	7	18	8213.33	49845.71	2.60	4.97E-06
**A0A075B6H9**	**IGLV4-69**	5	16	2256.94	13650.19	2.60	3.69E-06
**P50148**	**GNAQ**	11	18	24303.25	146163.25	2.59	1.70E-03
**P27105**	**STOM**	11	18	37529.01	222634.98	2.57	1.77E-05
**P08962**	**CD63**	11	18	15509.93	92003.89	2.57	3.55E-06

**P12429**	**ANXA3**	10	18	22349.24	131073.95	2.55	5.44E-08
**P01876**	**IGHA1**	10	18	104091.34	608814.34	2.55	2.98E-05
**O96009**	**NAPSA**	5	17	12593.01	73607.35	2.55	3.40E-04
**P12277**	**CKB**	7	17	9575.21	55804.00	2.54	1.60E-05
**P53801**	**PTTG1IP**	11	18	9615.09	55949.77	2.54	3.16E-04
**P48735**	**IDH2**	9	17	13746.84	79984.05	2.54	7.58E-05
**P09960**	**LTA4H**	10	15	8967.09	52153.96	2.54	4.00E-04
**P05155**	**SERPING1**	8	17	11641.75	67648.52	2.54	3.19E-06
**O00194**	**RAB27B**	9	18	19029.68	109451.07	2.52	4.58E-04
**P01009**	**SERPINA1**	10	17	18557.37	106177.89	2.52	6.17E-07
**O15143**	**ARPC1B**	7	16	7774.45	44396.87	2.51	1.08E-05
**P01889**	**HLA-B**	7	15	11863.51	65992.81	2.48	1.22E-05
**P63104**	**YWHAZ**	11	18	16714.93	92909.72	2.47	1.55E-05
**Q9NZM1**	**MYOF**	7	18	7149.40	39194.06	2.45	3.91E-02
**P08174**	**CD55**	11	18	29733.75	162760.11	2.45	1.65E-03
**P68371**	**TUBB4B**	10	17	19836.95	107454.23	2.44	6.20E-08
**P62805**	**H4C1**	8	18	17595.07	95137.14	2.43	7.03E-06
**P01834**	**IGKC**	10	17	132258.41	711268.69	2.43	9.61E-07
**P31947**	**SFN**	9	17	7879.59	42118.18	2.42	1.51E-05
**P01700**	**IGLV1-47**	9	17	8025.41	42545.29	2.41	3.25E-04
**P62258**	**YWHAE**	10	18	16427.53	86222.64	2.39	5.49E-04
**Q8NFJ5**	**GPRC5A**	11	18	157392.69	825460.95	2.39	1.58E-03
**A0A075B6K5**	**IGLV3-9**	5	17	14151.47	74180.31	2.39	3.68E-06
**P01619**	**IGKV3-20**	10	18	51210.51	266763.89	2.38	7.33E-06
**P06733**	**ENO1**	11	18	15368.58	79603.69	2.37	2.10E-04
**Q8WXI7**	**MUC16**	10	18	10862.81	55831.94	2.36	2.81E-08
**Q8N5I2**	**ARRDC1**	8	17	22419.06	114076.60	2.35	5.61E-03
**Q8IWA5**	**SLC44A2**	10	18	20795.94	105384.62	2.34	2.42E-03
**P01780**	**IGHV3-7**	3	17	7295.57	36808.86	2.33	2.66E-08
**Q8TE68**	**EPS8L1**	10	18	10312.67	52017.07	2.33	1.81E-04
**P09525**	**ANXA4**	11	18	33768.91	169958.71	2.33	1.26E-08
**Q8WUM4**	**PDCD6IP**	11	18	26746.72	134175.35	2.33	5.03E-04
**P10909**	**CLU**	10	17	19270.41	96570.88	2.33	1.89E-05
**P04229**	**HLA-DRB1**	11	18	40249.20	200596.34	2.32	1.13E-04
**P56856**	**CLDN18**	11	18	15873.32	77960.40	2.30	1.50E-02
**Q7Z404**	**TMC4**	11	18	20462.98	100365.20	2.29	3.79E-04
**P09211**	**GSTP1**	11	18	27730.71	133975.53	2.27	1.46E-05

**Q9P0V3**	**SH3BP4**	7	15	3842.50	18481.67	2.27	1.39E-05
**P31949**	**S100A11**	11	18	26651.04	127748.32	2.26	3.98E-07
**P06703**	**S100A6**	11	18	38655.92	184707.90	2.26	2.68E-08
**P03973**	**SLPI**	5	17	4688.81	22392.90	2.26	1.26E-13
**O95716**	**RAB3D**	9	17	13637.39	64878.80	2.25	1.47E-06
**Q9NZN4**	**EHD2**	11	17	20721.72	98248.66	2.25	3.79E-03
**P61586**	**RHOA**	11	18	8250.89	39061.32	2.24	1.65E-04
**P04075**	**ALDOA**	10	17	10812.94	51052.81	2.24	2.45E-06
**Q99828**	**CIB1**	11	18	34336.21	159933.63	2.22	1.43E-04
**O15162**	**PLSCR1**	8	17	9080.09	42281.86	2.22	1.01E-05
**P63000**	**RAC1**	11	18	38242.25	177773.48	2.22	3.79E-04
**P40199**	**CEACAM6**	9	17	32876.32	151667.47	2.21	2.20E-06
**Q9H6S3**	**EPS8L2**	11	18	33196.38	151961.98	2.19	6.23E-04
**P35241**	**RDX**	11	18	37544.99	171410.53	2.19	1.18E-03
**P62879**	**GNB2**	11	18	49693.78	226848.38	2.19	4.88E-04
**P68363**	**TUBA1B**	11	18	21023.61	95445.04	2.18	1.94E-07
**P51148**	**RAB5C**	11	18	20477.42	92151.90	2.17	3.90E-04
**O43657**	**TSPAN6**	11	18	13070.97	58770.07	2.17	8.22E-05
**P01714**	**IGLV3-19**	9	17	50323.18	225965.66	2.17	4.31E-03
**P37802**	**TAGLN2**	8	16	9650.68	42913.70	2.15	3.87E-06
**Q9UL25**	**RAB21**	10	15	12571.08	55740.97	2.15	2.05E-02
**Q9UQB8**	**BAIAP2**	11	18	22530.50	99199.58	2.14	8.46E-03
**P54707**	**ATP12A**	7	16	4296.72	18892.62	2.14	7.47E-06
**P61981**	**YWHAG**	9	16	5962.88	26163.43	2.13	1.01E-04
**P08758**	**ANXA5**	11	18	98597.41	429615.39	2.12	1.46E-09
**P29992**	**GNA11**	11	18	26232.09	114218.69	2.12	1.32E-03
**P53990**	**IST1**	11	17	26068.10	112941.95	2.12	1.13E-04
**P10301**	**RRAS**	11	18	34829.68	150776.27	2.11	1.03E-03
**Q08722**	**CD47**	8	17	30198.35	128907.37	2.09	3.36E-03
**P62834**	**RAP1A**	11	18	29068.07	123974.39	2.09	1.37E-02
**Q5VW32**	**BROX**	10	17	17442.09	74264.11	2.09	5.03E-04
**O00299**	**CLIC1**	11	18	35223.40	149618.16	2.09	1.69E-04
**Q13228**	**SELENBP1**	11	18	17378.20	73264.34	2.08	1.64E-03
**P04899**	**GNAI2**	11	18	23756.46	99399.61	2.06	3.15E-04
**P15941**	**MUC1**	11	18	114627.77	478975.14	2.06	1.57E-02
**P15311**	**EZR**	11	18	30073.13	125195.61	2.06	4.51E-04
**P20073**	**ANXA7**	11	18	18253.86	75602.72	2.05	2.65E-09
**O43795**	**MYO1B**	9	18	14574.47	60225.10	2.05	4.60E-03
**P46940**	**IQGAP1**	9	17	12993.10	53564.80	2.04	4.17E-03
**Q09666**	**AHNAK**	10	18	7817.22	32087.61	2.04	6.14E-04
**O95833**	**CLIC3**	11	17	18765.46	76966.06	2.04	1.33E-02

**P01860**	**IGHG3**	9	17	29004.48	118809.42	2.03	4.15E-04
**P62820**	**RAB1A**	11	17	9962.79	40660.68	2.03	3.58E-05
**Q15599**	**SLC9A3 R2**	11	18	16858.82	68124.82	2.01	1.96E-02
**P26038**	**MSN**	11	18	38679.39	155342.17	2.01	2.59E-02
**O15484**	**CAPN5**	6	15	8046.76	32311.99	2.01	2.11E-05
**P69905**	**HBA1**	11	18	45922.10	183957.98	2.00	1.71E-07
**O94832**	**MYO1D**	9	18	12365.91	49199.13	1.99	5.87E-04
**P61026**	**RAB10**	11	18	16708.75	66330.65	1.99	2.61E-04
**P60953**	**CDC42**	11	18	40637.51	160929.52	1.99	6.78E-03
**P48509**	**CD151**	10	18	18610.87	73690.78	1.99	1.77E-04
**P16050**	**ALOX15**	9	18	11219.94	44397.20	1.98	6.02E-08
**P13489**	**RNH1**	7	17	10791.12	42675.17	1.98	2.90E-03
**Q66K66**	**TMEM1 98**	10	17	98494.44	388132.66	1.98	7.10E-06
**P62917**	**RPL8**	5	16	5953.43	23333.68	1.97	2.73E-03
**P63261**	**ACTG1**	11	18	71222.51	279098.66	1.97	5.57E-06
**Q96FN4**	**CPNE2**	10	18	21494.68	83385.45	1.96	4.78E-03
**Q7LBR1**	**CHMP1 B**	11	17	16351.36	63330.03	1.95	3.92E-04
**P0C0L5**	**C4B**	6	15	10165.03	39283.80	1.95	9.51E-06
**B9A064**	**IGLL5**	9	17	23727.99	91613.86	1.95	2.78E-05
**P42685**	**FRK**	6	16	11262.48	43261.50	1.94	3.85E-04
**O43633**	**CHMP2 A**	6	15	22306.88	85270.74	1.93	1.29E-04
**P36969**	**GPX4**	9	16	11080.92	42307.10	1.93	4.10E-03
**O95436**	**SLC34A2**	11	18	51206.74	193777.38	1.92	1.53E-02
**P06744**	**GPI**	9	17	10929.04	41206.30	1.91	7.71E-05
**P07948**	**LYN**	8	16	14350.07	53951.01	1.91	1.35E-03
**P08134**	**RHOC**	11	18	23325.28	87108.07	1.90	7.25E-03
**A0A0B4J 1X5**	**IGHV3-74**	6	17	57829.35	215403.36	1.90	1.85E-04
**P27487**	**DPP4**	10	16	19310.51	71802.37	1.89	1.39E-03
**P07900**	**HSP90A A1**	8	17	21923.80	81276.92	1.89	1.71E-07
**P11234**	**RALB**	11	17	13874.64	50759.09	1.87	2.11E-03
**P12931**	**SRC**	11	18	21996.82	80346.35	1.87	3.76E-04
**Q6UXY8**	**TMC5**	11	18	27238.48	98524.18	1.85	1.86E-04
**Q9Y624**	**F11R**	8	15	19913.28	71569.84	1.85	2.23E-04
**Q99829**	**CPNE1**	6	15	10782.83	38577.74	1.84	8.05E-03
**P07737**	**PFN1**	7	17	18936.98	67616.36	1.84	9.26E-05
**Q9UN76**	**SLC6A14**	11	18	15337.06	54379.51	1.83	1.15E-02
**P27216**	**ANXA13**	9	17	15599.59	55257.43	1.82	3.32E-07
**Q9BW04**	**SARG**	11	18	12820.56	45244.35	1.82	2.94E-02
**P37837**	**TALDO1**	6	16	7859.01	27577.84	1.81	2.42E-09
**P61006**	**RAB8A**	8	16	11045.12	38745.22	1.81	1.60E-04

**P13473**	**LAMP2**	7	16	14340.00	50089.60	1.80	4.79E-02
**Q8N392**	**ARHGAP 18**	7	15	10689.65	37270.24	1.80	1.49E-03
**P17931**	**LGALS3**	11	18	32974.13	114027.16	1.79	6.72E-06
**P60174**	**TPI1**	10	18	18668.41	64554.30	1.79	7.59E-05
**P50995**	**ANXA11**	11	18	39552.10	136339.08	1.79	6.34E-07
**P06396**	**GSN**	9	17	15353.68	52630.48	1.78	5.64E-05
**P29508**	**SERPINB 3**	9	18	11651.25	39850.70	1.77	4.90E-06
**O43490**	**PROM1**	11	18	18103.77	61705.17	1.77	1.77E-04
**Q96TA1**	**NIBAN2**	8	17	12295.45	41906.76	1.77	3.16E-02
**Q9HD42**	**CHMP1 A**	9	16	6350.41	21600.37	1.77	2.05E-02
**Q96AQ2**	**TMEM1 25**	11	17	20909.52	70614.69	1.76	1.64E-03
**Q15907**	**RAB11B**	11	18	22589.01	76226.38	1.75	8.43E-04
**Q06830**	**PRDX1**	11	18	27887.87	93115.86	1.74	3.00E-06
**P31025**	**LCN1**	4	16	13722.60	45686.12	1.74	2.80E-12
**P13987**	**CD59**	11	18	109809.5 5	363903.25	1.73	6.08E-04
**P62873**	**GNB1**	11	18	42307.91	140169.79	1.73	3.83E-03
**P21926**	**CD9**	11	18	185367.5 8	612304.22	1.72	1.47E-02
**P04406**	**GAPDH**	11	18	26518.46	87466.70	1.72	6.68E-08
**P27701**	**CD82**	10	18	13925.98	45828.48	1.72	2.82E-03
**P08263**	**GSTA1**	11	18	36266.80	118860.99	1.71	9.45E-03
**P11142**	**HSPA8**	11	18	19737.46	64650.77	1.71	5.50E-05
**P29401**	**TKT**	11	17	11629.85	37948.06	1.71	4.87E-06
**Q9UBV8**	**PEF1**	11	15	14307.71	46582.54	1.70	6.19E-04
**P61225**	**RAP2B**	5	15	19284.14	62521.65	1.70	2.81E-02
**Q6P9B6**	**MEAK7**	7	15	3547.42	11494.87	1.70	1.57E-04
**Q04917**	**YWHAH**	8	16	11817.96	38135.22	1.69	7.88E-04
**A0A0B4J 1U7**	**IGHV6-1**	6	17	12359.08	39865.50	1.69	4.88E-04
**P05362**	**ICAM1**	10	18	66720.85	213482.80	1.68	2.33E-02
**P21980**	**TGM2**	8	18	21517.50	68409.20	1.67	6.31E-05
**P22314**	**UBA1**	6	16	9476.93	30085.24	1.67	3.30E-04
**P01859**	**IGHG2**	7	18	73795.10	233811.78	1.66	3.90E-04
**P52566**	**ARHGDI B**	8	17	9417.74	29703.43	1.66	3.03E-04
**P02774**	**GC**	7	16	10640.64	33295.22	1.65	5.00E-06
**P59998**	**ARPC4**	4	16	15777.10	49348.93	1.65	1.24E-02
**P13797**	**PLS3**	9	18	19923.98	62256.30	1.64	4.60E-05
**Q9UPU7**	**TBC1D2 B**	4	17	15917.34	49367.85	1.63	4.13E-09
**Q9P121**	**NTM**	7	15	22670.29	69795.49	1.62	6.20E-08
**O60488**	**ACSL4**	6	16	14449.99	44337.91	1.62	2.65E-02

**P63218**	**GNG5**	11	18	16124.62	48624.24	1.59	2.05E-02
**O75131**	**CPNE3**	11	18	21718.44	65312.46	1.59	2.11E-05
**Q96IU4**	**ABHD14 B**	11	18	10971.33	32972.57	1.59	9.32E-04
**P61158**	**ACTR3**	8	16	15772.99	47378.13	1.59	1.69E-04
**P04440**	**HLA-DPB1**	11	17	17956.06	53776.77	1.58	1.49E-02
**P00738**	**HP**	9	17	32872.28	98180.29	1.58	1.97E-02
**P30740**	**SERPINB 1**	6	17	16071.22	47961.70	1.58	4.90E-06
**Q9NRD9**	**DUOX1**	8	16	10821.08	32270.91	1.58	1.05E-04
**P13796**	**LCP1**	5	17	13529.89	39965.17	1.56	1.35E-05
**Q9UBI6**	**GNG12**	11	18	22287.55	65685.57	1.56	2.74E-03
**A0A0C4D H38**	**IGHV5-51**	6	17	15848.94	46658.02	1.56	8.06E-08
**P12814**	**ACTN1**	6	17	14611.46	42763.42	1.55	5.96E-05
**Q96EY5**	**MVB12 A**	6	16	10885.72	31740.80	1.54	1.18E-05
**Q96CX2**	**KCTD12**	10	16	14083.17	41040.31	1.54	4.85E-03
**Q9H1C7**	**CYSTM1**	10	18	166260.0 8	482096.00	1.54	6.08E-05
**P30408**	**TM4SF1**	10	17	38941.66	112349.79	1.53	6.53E-03
**Q9NRX4**	**PHPT1**	8	17	14646.36	42034.81	1.52	2.95E-05
**P18085**	**ARF4**	6	16	32503.44	92933.91	1.52	1.73E-02
**P19823**	**ITIH2**	7	16	13629.52	38903.78	1.51	6.46E-04
**P02794**	**FTH1**	6	16	9818.72	27673.83	1.49	1.99E-07
**O75083**	**WDR1**	9	18	15648.85	43721.20	1.48	8.23E-04
**P01903**	**HLA-DRA**	11	18	44887.55	123818.12	1.46	1.28E-02
**P02654**	**APOC1**	5	15	12229.90	33197.90	1.44	1.41E-06
**Q9UGT4**	**SUSD2**	11	18	55725.05	149716.23	1.43	9.80E-03
**P52943**	**CRIP2**	7	16	13175.37	35381.66	1.43	4.96E-02
**P30626**	**SRI**	10	17	29242.57	78516.34	1.42	1.29E-05
**Q9UK41**	**VPS28**	6	16	16498.35	44035.29	1.42	4.89E-03
**O95164**	**UBL3**	11	18	17744.20	47128.81	1.41	1.85E-02
**O14745**	**SLC9A3 R1**	11	18	22978.96	60660.22	1.40	4.82E-05
**Q5T700**	**LDLRAD 1**	5	15	6230.05	16347.06	1.39	9.51E-06
**P05023**	**ATP1A1**	7	16	15504.07	40521.10	1.39	1.41E-04
**P02649**	**APOE**	4	17	22313.33	57849.44	1.37	4.06E-06
**Q96A22**	**C11orf5 2**	11	18	10854.44	28109.11	1.37	1.37E-03
**Q00610**	**CLTC**	7	16	10898.87	27823.58	1.35	1.49E-06
**Q99732**	**LITAF**	8	17	20630.95	52508.45	1.35	1.39E-02
**O75340**	**PDCD6**	11	17	44683.05	113227.64	1.34	3.80E-03
**Q9H0E2**	**TOLLIP**	11	17	15470.59	39193.27	1.34	1.60E-03

**Q96FZ7**	**CHMP6**	7	15	25149.75	63404.02	1.33	3.92E-04
**O00391**	**QSOX1**	3	17	6744.49	16856.35	1.32	2.11E-05
**Q9HCY8**	**S100A14**	6	17	10572.70	26087.99	1.30	2.39E-04
**Q15836**	**VAMP3**	8	15	6154.95	15173.25	1.30	8.06E-05
**P55064**	**AQP5**	8	17	32881.35	80057.72	1.28	2.40E-04
**P00558**	**PGK1**	9	18	23084.14	56039.69	1.28	6.57E-05
**O94760**	**DDAH1**	8	16	24108.00	58290.50	1.27	4.72E-03
**O00161**	**SNAP23**	8	16	18515.89	44678.53	1.27	1.44E-03
**P43353**	**ALDH3B1**	11	18	63999.51	152997.40	1.26	1.59E-03
**P06312**	**IGKV4-1**	7	17	13886.08	33175.85	1.26	3.67E-06
**Q99102**	**MUC4**	11	18	29780.55	70430.51	1.24	5.13E-08
**Q92485**	**SMPDL3B**	7	15	37975.19	89487.79	1.24	9.14E-04
**P62140**	**PPP1CB**	7	17	14526.09	34102.03	1.23	2.38E-02
**O60635**	**TSPAN1**	11	18	81599.34	189403.99	1.21	5.12E-03
**P62745**	**RHOB**	8	15	28836.00	66916.03	1.21	3.67E-02
**P61160**	**ACTR2**	6	16	17130.95	39225.84	1.20	1.14E-04
**P50502**	**ST13**	5	15	16269.45	37015.52	1.19	1.86E-03
**P61020**	**RAB5B**	8	16	13107.31	29750.51	1.18	2.40E-03
**Q96KP4**	**CNDP2**	11	18	24129.26	54653.83	1.18	4.40E-05
**P23528**	**CFL1**	11	18	20968.79	47483.26	1.18	5.52E-04
**Q99816**	**TSG101**	11	18	30495.02	68322.19	1.16	1.50E-02
**P68402**	**PAFAH1B2**	5	15	5301.70	11820.10	1.16	8.91E-03
**P47929**	**LGALS7**	5	16	7859.20	17441.95	1.15	1.79E-03
**Q9H4A4**	**RNPEP**	7	17	12906.31	28580.42	1.15	1.74E-04
**Q8IYJ3**	**SYTL1**	5	16	18964.66	41770.30	1.14	4.80E-03
**P28062**	**PSMB8**	5	15	10979.29	24139.32	1.14	6.17E-07
**P28838**	**LAP3**	6	16	11366.34	24905.56	1.13	5.43E-06
**P04196**	**HRG**	4	16	16816.94	36752.39	1.13	2.19E-06
**Q9BV40**	**VAMP8**	9	17	14067.39	30673.41	1.12	7.59E-05
**P21333**	**FLNA**	5	15	11507.93	24929.67	1.12	1.48E-05
**P60903**	**S100A10**	10	16	34386.15	74295.97	1.11	3.09E-03
**P02743**	**APCS**	6	15	12650.79	27323.25	1.11	1.44E-05
**P00450**	**CP**	8	17	19040.43	40592.42	1.09	9.80E-03
**P68871**	**HBB**	10	18	73334.61	156015.36	1.09	2.02E-06
**P55072**	**VCP**	6	17	19553.31	41103.34	1.07	3.40E-06
**P02792**	**FTL**	4	17	15797.54	33011.32	1.06	9.82E-07
**P19075**	**TSPAN8**	8	17	13506.14	27948.92	1.05	1.39E-05
**Q86TJ2**	**TADA2B**	8	16	27075.97	54630.58	1.01	6.79E-03
**P52209**	**PGD**	9	17	16598.13	32941.12	0.99	3.13E-04
**Q05655**	**PRKCD**	8	17	12697.94	25193.51	0.99	2.94E-02
**O75695**	**RP2**	9	16	11527.00	22763.86	0.98	1.52E-02

**P60981**	**DSTN**	11	18	18195.63	35455.16	0.96	1.26E-02
**Q53GD3**	**SLC44A4**	11	18	58689.45	114014.26	0.96	4.61E-03
**P13693**	**TPT1**	6	15	7379.57	14279.25	0.95	1.64E-04
**P02679**	**FGG**	9	18	19049.89	36785.47	0.95	4.87E-05
**Q9HAV0**	**GNB4**	6	15	17052.29	32802.72	0.94	9.71E-03
**P00338**	**LDHA**	8	17	50211.80	96580.37	0.94	1.58E-04
**P01008**	**SERPINC 1**	9	17	18293.73	35110.18	0.94	6.67E-03
**Q969L2**	**MAL2**	7	18	71893.75	137625.93	0.94	2.25E-02
**P19440**	**GGT1**	7	16	32644.91	62212.17	0.93	4.48E-05
**P40925**	**MDH1**	7	17	26974.85	51104.63	0.92	2.23E-04
**P30838**	**ALDH3A 1**	8	17	15440.21	29149.88	0.92	6.44E-05
**Q6P6B1**	**ERICH5**	7	16	8225.02	15365.33	0.90	8.40E-04
**Q13938**	**CAPS**	10	18	36154.96	66557.46	0.88	1.01E-02
**P30044**	**PRDX5**	11	18	43875.85	80285.98	0.87	2.90E-03
**O95837**	**GNA14**	8	16	16338.84	29873.07	0.87	1.35E-03
**P04632**	**CAPNS1**	4	16	20710.09	37861.63	0.87	2.97E-04
**P15104**	**GLUL**	5	17	13197.93	23766.24	0.85	2.16E-03
**Q9Y490**	**TLN1**	5	15	7858.96	14113.02	0.84	2.55E-04
**P67936**	**TPM4**	4	17	8135.66	14588.83	0.84	6.89E-04
**P61019**	**RAB2A**	7	15	30923.36	54730.82	0.82	3.57E-02
**P06727**	**APOA4**	7	15	26117.18	46140.74	0.82	2.19E-06
**Q99497**	**PARK7**	8	17	19852.57	34997.25	0.82	2.19E-02
**Q96NY7**	**CLIC6**	10	18	16749.20	29516.32	0.82	1.65E-03
**P11686**	**SFTPC**	6	15	13034.48	22802.03	0.81	1.59E-03
**P30041**	**PRDX6**	10	18	52847.21	90609.85	0.78	5.29E-03
**P62070**	**RRAS2**	6	15	22808.69	38608.21	0.76	2.10E-02
**Q9H4G4**	**GLIPR2**	10	17	59550.92	100706.16	0.76	2.31E-02
**P50395**	**GDI2**	8	18	32014.07	54059.42	0.76	1.70E-02
**P25787**	**PSMA2**	5	15	7589.83	12669.69	0.74	3.07E-05
**Q8TAV4**	**STOML3**	5	16	11154.76	18538.25	0.73	1.94E-03
**O43866**	**CD5L**	6	16	10115.47	16718.95	0.72	4.98E-04
**P62826**	**RAN**	6	17	26447.18	43232.98	0.71	2.49E-05
**P00352**	**ALDH1A 1**	10	18	38611.22	63094.09	0.71	2.90E-03
**Q16555**	**DPYSL2**	5	17	25386.52	41430.84	0.71	1.41E-02
**P10599**	**TXN**	11	18	36254.34	58879.00	0.70	8.95E-04
**Q9P265**	**DIP2B**	5	15	23425.71	36759.27	0.65	1.02E-02
**P14923**	**JUP**	9	18	11542.93	18000.40	0.64	1.26E-03
**P01042**	**KNG1**	5	16	17447.20	27087.80	0.63	1.16E-05
**P02765**	**AHSG**	5	16	17509.84	27092.54	0.63	8.23E-06
**Q96AX2**	**RAB37**	5	15	21421.55	32766.93	0.61	1.81E-04
**P05107**	**ITGB2**	5	16	28874.70	43729.40	0.60	3.57E-05
**P39019**	**RPS19**	5	15	11093.88	16784.83	0.60	8.91E-04
**P61106**	**RAB14**	6	15	16345.83	24569.65	0.59	4.38E-03

**P02538**	**KRT6A**	11	18	37242.40	55931.05	0.59	4.60E-03
**P01602**	**IGKV1-5**	4	17	251740.7<br>3	376759.38	0.58	3.68E-04
**P04040**	**CAT**	9	17	20386.73	30334.88	0.57	3.31E-02
**P13726**	**F3**	5	16	17478.07	26000.97	0.57	2.19E-02
**P61626**	**LYZ**	10	18	14816.62	21984.74	0.57	4.49E-04
**P22735**	**TGM1**	8	15	13835.13	20452.85	0.56	2.44E-03
**P57735**	**RAB25**	6	15	33663.89	49688.51	0.56	1.27E-03
**Q9NQ79**	**CRTAC1**	6	15	12609.40	18585.27	0.56	1.53E-03
**P19971**	**TYMP**	4	17	27943.25	40700.92	0.54	1.62E-04
**P15924**	**DSP**	11	18	12624.01	18015.10	0.51	1.69E-04
**Q9Y230**	**RUVBL2**	6	15	15077.32	21453.85	0.51	4.66E-05
**P25786**	**PSMA1**	5	15	14473.09	20514.78	0.50	6.18E-05
**P78417**	**GSTO1**	8	17	31401.39	44087.15	0.49	2.38E-02
**P80723**	**BASP1**	11	17	7107.88	9953.05	0.49	6.46E-04
**Q53TN4**	**CYBRD1**	10	18	57418.88	79104.88	0.46	3.29E-02
**P16152**	**CBR1**	6	17	28795.03	39255.45	0.45	1.33E-02
**O43707**	**ACTN4**	5	18	18423.95	24394.44	0.40	7.77E-03
**P07384**	**CAPN1**	4	17	27039.93	35689.26	0.40	2.64E-02
**P02749**	**APOH**	5	15	18509.75	24414.74	0.40	2.66E-07
**P17655**	**CAPN2**	5	15	30649.91	40163.85	0.39	9.51E-05
**Q8N9U0**	**TC2N**	6	16	20795.12	26884.48	0.37	1.24E-02
**P02787**	**TF**	9	17	28135.60	36182.37	0.36	4.30E-05
**P22528**	**SPRR1B**	9	18	13255.72	16958.28	0.36	1.02E-02
**P13647**	**KRT5**	11	18	32413.68	41313.18	0.35	3.04E-03
**P02533**	**KRT14**	11	18	45174.99	56907.23	0.33	6.68E-03
**P05090**	**APOD**	4	16	10614.51	12999.29	0.29	2.28E-07
**P01031**	**C5**	5	16	15572.13	18423.59	0.24	1.17E-04
**P26447**	**S100A4**	4	16	30173.02	35636.67	0.24	3.05E-02
**P36578**	**RPL4**	4	17	10327.24	9882.69	-<br>0.06	2.80E-05
**P29966**	**MARCKS**	8	16	9553.06	9052.93	-<br>0.08	8.38E-03
**O75558**	**STX11**	5	15	22564.99	21366.40	-<br>0.08	1.45E-02
**Q8IWU2**	**LMTK2**	11	17	35855.73	33724.52	-<br>0.09	7.93E-05
**P00751**	**CFB**	8	17	26330.92	24645.16	-<br>0.10	1.45E-03
**P08779**	**KRT16**	10	18	20922.58	18846.81	-<br>0.15	2.39E-02
**Q04695**	**KRT17**	10	18	20938.46	18010.64	-<br>0.22	2.86E-02
**P07741**	**APRT**	5	16	59865.34	50852.88	-<br>0.24	2.96E-03
**P14550**	**AKR1A1**	5	16	22440.14	18047.11	-<br>0.31	4.12E-03

**P05089**	**ARG1**	9	18	24670.92	18064.68	-0.45	7.78E-03
**P12273**	**PIP**	7	18	30829.10	22128.42	-0.48	2.38E-02
**P0C672**	**TSPAN19**	6	16	13619.85	9258.51	-0.56	4.53E-02
**P50238**	**CRIP1**	4	17	37556.03	25055.73	-0.58	5.38E-04
**P35237**	**SERPINB6**	3	17	54993.51	34332.82	-0.68	2.47E-02
**P00325**	**ADH1B**	7	16	31335.92	16022.30	-0.97	3.54E-02
**P51674**	**GPM6A**	7	17	154750.38	65956.73	-1.23	2.08E-03
**P19827**	**ITIH1**	10	16	262254.70	17411.42	-3.91	7.19E-05
**Q14376**	**GALE**	6	16	1017610.56	19860.06	-5.68	2.78E-02
**Q99758**	**ABCA3**	9	16	12059.83	100308.96	3.06	FDR > 0.05
**P59665;P59666**	**DEFA1;DEFA3**	5	16	15123.16	111356.39	2.88	FDR > 0.05
**P84095**	**RHOG**	9	16	8280.25	59987.02	2.86	FDR > 0.05
**P0DOY2;P0DOY3**	**IGLC2;IGLC3**	11	18	89722.35	562527.41	2.65	FDR > 0.05
**Q14254**	**FLOT2**	11	17	10000.46	61452.38	2.62	FDR > 0.05
**Q92817**	**EVPL**	10	18	7021.76	39300.40	2.48	FDR > 0.05
**A0A0A0MRZ8;P04433**	**IGKV3D-11;IGKV3-11**	5	16	26030.35	143930.20	2.47	FDR > 0.05
**Q8IVT2**	**MISP**	7	13	2431.86	12885.92	2.41	FDR > 0.05
**P0CG47;P0CG48;P62979;P62987**	**UBB;UBC;RPS27A;UBA52**	11	18	224072.98	1176804.63	2.39	FDR > 0.05
**Q9Y376**	**CAB39**	7	16	9227.99	47956.23	2.38	FDR > 0.05
**O60437**	**PPL**	10	17	10118.15	52126.39	2.37	FDR > 0.05
**Q9Y5K6**	**CD2AP**	7	13	5397.32	27342.34	2.34	FDR > 0.05
**P05186**	**ALPL**	11	18	17454.19	87806.54	2.33	FDR > 0.05
**Q99571**	**P2RX4**	9	15	18882.16	92201.49	2.29	FDR > 0.05
**Q16881**	**TXNRD1**	7	14	7047.13	33977.04	2.27	FDR > 0.05

**A0A0C4DH41;P01824;P01825;P06331;P0DP06;P0DP08**	**IGHV4-61;IGHV4-39;IGHV4-59;IGHV4-34;IGHV4-30-4;IGHV4-38-2**	7	17	14941.77	71994.64	2.27	FDR > 0.05
**O60603**	**TLR2**	10	14	7613.50	36315.76	2.25	FDR > 0.05
**P01023**	**A2M**	9	17	21314.71	95411.09	2.16	FDR > 0.05
**P13861**	**PRKAR2A**	11	18	14959.74	65652.36	2.13	FDR > 0.05
**Q9H4M9**	**EHD1**	9	17	18615.01	80081.63	2.11	FDR > 0.05
**Q9HAC8**	**UBTD1**	6	14	10672.17	44398.00	2.06	FDR > 0.05
**P68104;Q5VTE0**	**EEF1A1;EEF1A1P5**	10	18	26606.66	107544.22	2.02	FDR > 0.05
**Q9H223**	**EHD4**	10	18	16828.88	67239.45	2.00	FDR > 0.05
**Q12929**	**EPS8**	11	18	23832.20	95201.45	2.00	FDR > 0.05
**A0A075B6S2;A2NJV5**	**IGKV2D-29;IGKV2-29**	7	17	30675.55	113475.09	1.89	FDR > 0.05
**P06899;P23527;P33778;Q16778**	**HIST1H2BJ;HIST1H2BO;HIST1H2BB;HIST2H2BE**	7	17	11195.21	40990.21	1.87	FDR > 0.05
**P15144**	**ANPEP**	11	17	20200.89	73888.02	1.87	FDR > 0.05
**A0A1B0GWB2**	**PRRT1B**	9	13	10405.51	37305.61	1.84	FDR > 0.05
**P01768;P0DP03**	**IGHV3-30;IGHV3-30-5**	8	17	12091.48	43241.07	1.84	FDR > 0.05
**Q5HYA8**	**TMEM67**	8	13	6405.46	22706.98	1.83	FDR > 0.05
**P14384**	**CPM**	7	13	12704.83	44881.25	1.82	FDR > 0.05
**Q14108**	**SCARB2**	9	16	13305.31	45926.29	1.79	FDR > 0.05

**P27348**	**YWHAQ**	7	16	10032.72	34615.80	1.79	FDR > 0.05
**Q3B8N2; Q6DKI2**	**LGALS9 B;LGALS 9C**	7	16	13513.52	46184.88	1.77	FDR > 0.05
**O75955**	**FLOT1**	7	16	10344.42	35016.16	1.76	FDR > 0.05
**Q9UNF0**	**PACSIN2**	8	17	14941.73	49578.89	1.73	FDR > 0.05
**Q16348**	**SLC15A2**	8	17	22938.03	74399.30	1.70	FDR > 0.05
**Q12923**	**PTPN13**	7	17	14507.59	46734.77	1.69	FDR > 0.05
**P01706;P 01709**	**IGLV2-11;IGLV 2-8**	7	17	26579.83	85050.98	1.68	FDR > 0.05
**Q16651**	**PRSS8**	8	16	19687.18	62987.21	1.68	FDR > 0.05
**Q969X1**	**TMBIM1**	11	18	65152.28	207497.05	1.67	FDR > 0.05
**P01920**	**HLA-DQB1**	7	14	34621.68	109745.70	1.66	FDR > 0.05
**P84077**	**ARF1**	10	18	37530.48	116903.63	1.64	FDR > 0.05
**P02671**	**FGA**	6	16	6234.06	19385.57	1.64	FDR > 0.05
**Q16777; Q6FI13**	**HIST2H2 AC;HIST 2H2AA3**	6	17	24393.89	75363.59	1.63	FDR > 0.05
**P00568**	**AK1**	9	18	19510.59	60255.13	1.63	FDR > 0.05
**Q8IWL1; Q8IWL2**	**SFTPA2; SFTPA1**	8	17	24691.06	76104.78	1.62	FDR > 0.05
**P20339**	**RAB5A**	7	14	19420.53	59573.17	1.62	FDR > 0.05
**P61088**	**UBE2N**	7	17	19158.43	56378.89	1.56	FDR > 0.05
**P16671**	**CD36**	10	17	35904.85	105248.47	1.55	FDR > 0.05
**Q9NY47**	**CACNA2 D2**	11	17	35534.54	103903.88	1.55	FDR > 0.05
**Q9UHR4**	**BAIAP2L 1**	11	16	14496.24	42240.50	1.54	FDR > 0.05
**P09543**	**CNP**	8	18	18291.78	53247.09	1.54	FDR > 0.05
**P04439**	**HLA-A**	9	15	23105.61	66913.24	1.53	FDR > 0.05
**O95336**	**PGLS**	6	14	16444.38	47618.33	1.53	FDR > 0.05
**P0DMV8; P0DMV9**	**HSPA1A ;HSPA1B**	11	18	24940.48	71946.93	1.53	FDR > 0.05

**P04745**	**AMY1A**	6	14	6320.83	18149.13	1.52	FDR > 0.05
**P62736;P 63267**	**ACTA2; ACTG2**	11	17	56062.05	160154.03	1.51	FDR > 0.05
**P01906**	**HLA-DQA2**	10	17	66085.93	187778.25	1.51	FDR > 0.05
**P63092; Q5JWF2**	**GNAS**	9	18	31290.54	87748.36	1.49	FDR > 0.05
**Q13277**	**STX3**	8	16	7367.73	20169.74	1.45	FDR > 0.05
**Q687X5**	**STEAP4**	10	18	38601.24	104020.80	1.43	FDR > 0.05
**Q9H7P6**	**MVB12 B**	8	12	17259.46	44916.14	1.38	FDR > 0.05
**Q9BUL8**	**PDCD10**	8	18	27960.93	71522.48	1.35	FDR > 0.05
**P35247**	**SFTPD**	11	16	163502.2 7	411820.36	1.33	FDR > 0.05
**Q9NQ84**	**GPRC5C**	10	18	37185.73	92193.30	1.31	FDR > 0.05
**P05091**	**ALDH2**	10	18	30555.76	75416.28	1.30	FDR > 0.05
**P35579**	**MYH9**	6	15	8059.60	19676.30	1.29	FDR > 0.05
**Q15833**	**STXBP2**	9	17	18423.26	43725.60	1.25	FDR > 0.05
**P21589**	**NT5E**	9	18	15914.30	37610.05	1.24	FDR > 0.05
**P0DP23; P0DP24; P0DP25**	**CALM1; CALM2; CALM3**	11	18	28852.01	67840.84	1.23	FDR > 0.05
**P36269**	**GGT5**	7	16	15743.11	36996.71	1.23	FDR > 0.05
**P49189**	**ALDH9A 1**	9	17	9984.97	23125.26	1.21	FDR > 0.05
**Q86YZ3**	**HRNR**	11	18	5600.38	12290.77	1.13	FDR > 0.05
**Q9NZN3**	**EHD3**	11	18	42753.61	93704.52	1.13	FDR > 0.05
**P29972**	**AQP1**	11	18	54385.01	119136.54	1.13	FDR > 0.05
**Q86YQ8**	**CPNE8**	11	18	22570.42	49128.04	1.12	FDR > 0.05
**P51153**	**RAB13**	7	14	23377.57	50783.01	1.12	FDR > 0.05
**Q15286**	**RAB35**	8	16	11134.85	23633.44	1.09	FDR > 0.05
**Q9Y696**	**CLIC4**	7	14	22099.51	46316.66	1.07	FDR > 0.05
**P12821**	**ACE**	6	17	24359.18	50898.48	1.06	FDR > 0.05

**Q9NP55**	**BPIFA1**	11	18	29035.38	60172.88	1.05	FDR > 0.05
**Q13404**	**UBE2V1**	6	15	19881.15	41057.23	1.05	FDR > 0.05
**P06737**	**PYGL**	6	16	9573.25	19693.28	1.04	FDR > 0.05
**Q01469**	**FABP5**	11	18	29847.81	60793.57	1.03	FDR > 0.05
**Q9H444**	**CHMP4B**	8	15	18187.10	36908.00	1.02	FDR > 0.05
**P04156**	**PRNP**	6	15	13812.44	27982.66	1.02	FDR > 0.05
**P32119**	**PRDX2**	9	18	25599.16	51676.52	1.01	FDR > 0.05
**P36405**	**ARL3**	6	14	17182.45	34481.56	1.00	FDR > 0.05
**P32856**	**STX2**	7	17	12066.13	24182.24	1.00	FDR > 0.05
**P09104**	**ENO2**	6	16	25461.08	50690.63	0.99	FDR > 0.05
**P09210**	**GSTA2**	7	17	25611.95	50939.55	0.99	FDR > 0.05
**Q9UQV4**	**LAMP3**	8	17	68660.44	135286.20	0.98	FDR > 0.05
**P61764**	**STXBP1**	8	17	20327.19	38091.49	0.91	FDR > 0.05
**P01011**	**SERPINA3**	7	17	17780.23	33224.50	0.90	FDR > 0.05
**Q9UQN3**	**CHMP2B**	7	17	42218.70	77354.13	0.87	FDR > 0.05
**Q562R1**	**ACTBL2**	9	18	115630.59	208423.04	0.85	FDR > 0.05
**P20036**	**HLA-DPA1**	10	16	45891.91	82003.16	0.84	FDR > 0.05
**P60709**	**ACTB**	7	16	54119.39	95228.04	0.82	FDR > 0.05
**Q9NZA1**	**CLIC5**	6	16	12351.91	21577.59	0.80	FDR > 0.05
**Q04828**	**AKR1C1**	9	18	26790.79	45966.16	0.78	FDR > 0.05
**Q4VNC1**	**ATP13A4**	5	15	19715.50	33676.43	0.77	FDR > 0.05
**P04264**	**KRT1**	11	18	72790.04	123676.29	0.76	FDR > 0.05
**O15551**	**CLDN3**	8	17	51977.00	87347.72	0.75	FDR > 0.05
**P02790**	**HPX**	4	17	18810.11	30629.91	0.70	FDR > 0.05
**Q9UKS6**	**PACSIN3**	6	14	14513.85	22966.47	0.66	FDR > 0.05

**Q9NZZ3**	**CHMP5**	8	15	26069.90	41200.04	0.66	FDR > 0.05
**O75351**	**VPS4B**	8	18	32594.08	51502.95	0.66	FDR > 0.05
**Q96QA5**	**GSDMA**	8	15	7960.67	12501.88	0.65	FDR > 0.05
**Q7L576**	**CYFIP1**	7	16	16477.73	25590.01	0.64	FDR > 0.05
**P51149**	**RAB7A**	10	16	47562.04	73430.76	0.63	FDR > 0.05
**P54920**	**NAPA**	6	16	23625.18	36271.44	0.62	FDR > 0.05
**Q9NP79**	**VTA1**	9	18	17977.80	27312.86	0.60	FDR > 0.05
**P35527**	**KRT9**	11	18	64002.72	97203.11	0.60	FDR > 0.05
**P50225**	**SULT1A1**	8	14	25702.73	38411.62	0.58	FDR > 0.05
**Q99536**	**VAT1**	5	16	40394.24	60285.86	0.58	FDR > 0.05
**Q86VE9**	**SERINC5**	9	15	22000.14	32386.82	0.56	FDR > 0.05
**P30086**	**PEBP1**	10	18	30261.74	44170.40	0.55	FDR > 0.05
**Q96PU5**	**NEDD4L**	6	14	13351.91	19452.29	0.54	FDR > 0.05
**Q9Y6E0**	**STK24**	8	17	27980.10	40284.47	0.53	FDR > 0.05
**P13645**	**KRT10**	11	18	137140.80	193906.54	0.50	FDR > 0.05
**Q9H2G2**	**SLK**	6	17	19349.13	27133.34	0.49	FDR > 0.05
**P08754**	**GNAI3**	8	17	41878.10	58582.82	0.48	FDR > 0.05
**O14817**	**TSPAN4**	8	13	30847.25	42884.66	0.48	FDR > 0.05
**Q14344**	**GNA13**	4	16	32767.03	45375.96	0.47	FDR > 0.05
**P04259**	**KRT6B**	11	18	86661.12	119932.21	0.47	FDR > 0.05
**Q3SY84**	**KRT71**	6	15	64085.96	88344.86	0.46	FDR > 0.05
**P53634**	**CTSC**	4	16	16462.46	22664.52	0.46	FDR > 0.05
**Q71RC9**	**SMIM5**	10	18	36489.30	50004.21	0.45	FDR > 0.05
**Q5VZK9**	**CARMIL1**	5	16	30968.25	42351.64	0.45	FDR > 0.05
**P49327**	**FASN**	6	14	20304.52	27683.85	0.45	FDR > 0.05

**P01111**	**NRAS**	6	17	44896.39	61042.96	0.44	FDR > 0.05
**P60033**	**CD81**	11	18	94397.16	126942.65	0.43	FDR > 0.05
**Q15517**	**CDSN**	8	18	23905.24	32018.26	0.42	FDR > 0.05
**Q96QR1**	**SCGB3A1**	8	17	21320.32	28022.41	0.39	FDR > 0.05
**P43652**	**AFM**	5	16	5674.03	7450.58	0.39	FDR > 0.05
**P63241**	**EIF5A**	8	16	19445.01	25432.05	0.39	FDR > 0.05
**O15393**	**TMPRSS2**	6	17	12924.07	16679.49	0.37	FDR > 0.05
**Q9Y2A7**	**NCKAP1**	7	16	22297.55	28519.96	0.36	FDR > 0.05
**Q6KB66**	**KRT80**	9	17	9026.91	11427.52	0.34	FDR > 0.05
**P35030**	**PRSS3**	5	16	18809.40	23772.15	0.34	FDR > 0.05
**P35908**	**KRT2**	11	18	66672.88	84023.61	0.33	FDR > 0.05
**P36871**	**PGM1**	6	16	19145.08	24021.41	0.33	FDR > 0.05
**Q08188**	**TGM3**	10	18	13727.93	17099.81	0.32	FDR > 0.05
**P07947**	**YES1**	4	16	31104.91	37712.22	0.28	FDR > 0.05
**P00966**	**ASS1**	7	17	20693.86	25009.98	0.27	FDR > 0.05
**P11413**	**G6PD**	6	15	25548.71	30846.40	0.27	FDR > 0.05
**Q5T749**	**KPRP**	11	18	18969.17	22810.38	0.27	FDR > 0.05
**P05787**	**KRT8**	11	18	24140.32	28816.03	0.26	FDR > 0.05
**Q9BW30**	**TPPP3**	11	17	29728.64	35424.61	0.25	FDR > 0.05
**P23284**	**PPIB**	6	15	11103.81	13198.62	0.25	FDR > 0.05
**Q9UN37**	**VPS4A**	7	17	22700.30	26820.52	0.24	FDR > 0.05
**P20930**	**FLG**	9	17	9080.15	10718.58	0.24	FDR > 0.05
**P51659**	**HSD17B4**	11	11	49947.62	58943.61	0.24	FDR > 0.05
**Q6Y7W6**	**GIGYF2**	10	18	141739.48	167108.52	0.24	FDR > 0.05
**Q13867**	**BLMH**	7	17	7532.69	8839.82	0.23	FDR > 0.05

**Q08554**	**DSC1**	7	17	11480.60	13469.20	0.23	FDR > 0.05
**P30043**	**BLVRB**	4	16	31829.60	37287.75	0.23	FDR > 0.05
**Q8N271**	**PROM2**	4	16	19757.03	23113.95	0.23	FDR > 0.05
**P04792**	**HSPB1**	8	18	39292.73	45770.78	0.22	FDR > 0.05
**P63096**	**GNAI1**	8	17	35270.78	40550.33	0.20	FDR > 0.05
**P16070**	**CD44**	9	16	28525.90	32355.83	0.18	FDR > 0.05
**P25788**	**PSMA3**	5	17	14428.81	16205.63	0.17	FDR > 0.05
**K7EJ46**	**SMIM22**	11	18	334074.47	370876.52	0.15	FDR > 0.05
**P22897**	**MRC1**	7	16	23628.57	26136.08	0.15	FDR > 0.05
**P52907**	**CAPZA1**	6	16	21025.65	22955.42	0.13	FDR > 0.05
**Q7Z794**	**KRT77**	11	18	29713.70	31245.58	0.07	FDR > 0.05
**P81605**	**DCD**	11	18	35901.46	37522.49	0.06	FDR > 0.05
**Q16851**	**UGP2**	6	14	25275.11	26398.21	0.06	FDR > 0.05
**Q08380**	**LGALS3BP**	11	18	573287.44	595587.78	0.06	FDR > 0.05
**P47756**	**CAPZB**	5	15	16574.06	17125.73	0.05	FDR > 0.05
**Q9C0H9**	**SRCIN1**	11	16	47677.99	49080.81	0.04	FDR > 0.05
**O15400**	**STX7**	6	17	58937.87	59875.86	0.02	FDR > 0.05
**P09972**	**ALDOC**	8	17	39085.68	38527.21	-0.02	FDR > 0.05
**P07477**	**PRSS1**	11	18	151804.89	146445.23	-0.05	FDR > 0.05
**P55786**	**NPEPPS**	6	18	21020.51	20158.70	-0.06	FDR > 0.05
**Q02413**	**DSG1**	11	18	15016.69	14262.21	-0.07	FDR > 0.05
**P31944**	**CASP14**	7	16	12694.90	12043.31	-0.08	FDR > 0.05
**P09417**	**QDPR**	10	18	36741.83	34748.39	-0.08	FDR > 0.05
**P05388; Q8NHW5**	**RPLP0;RPLP0P6**	11	18	103194.09	96608.43	-0.10	FDR > 0.05
**P20039**	**HLA-DRB1**	10	17	96140.01	89935.39	-0.10	FDR > 0.05

**Q5D862**	**FLG2**	10	18	7190.09	6717.31	-0.10	FDR > 0.05
**Q9UHI7**	**SLC23A1**	6	15	12422.09	11525.97	-0.11	FDR > 0.05
**P22532;P35325**	**SPRR2D; SPRR2B**	11	17	37228.57	34396.40	-0.11	FDR > 0.05
**Q99456**	**KRT12**	11	18	608189.69	561803.13	-0.11	FDR > 0.05
**P00441**	**SOD1**	6	14	29022.89	26777.22	-0.12	FDR > 0.05
**Q00796**	**SORD**	9	17	23634.43	21757.35	-0.12	FDR > 0.05
**Q12913**	**PTPRJ**	6	15	42924.96	38724.52	-0.15	FDR > 0.05
**P27797**	**CALR**	5	15	3617.11	3262.29	-0.15	FDR > 0.05
**P13646**	**KRT13**	11	18	134465.89	118157.68	-0.19	FDR > 0.05
**P19013**	**KRT4**	9	17	17459.71	15263.89	-0.19	FDR > 0.05
**P25311**	**AZGP1**	5	16	15592.29	13314.55	-0.23	FDR > 0.05
**P07339**	**CTSD**	8	18	29676.41	25155.75	-0.24	FDR > 0.05
**Q9NZT1**	**CALML5**	11	18	56343.72	47041.94	-0.26	FDR > 0.05
**Q14CZ7**	**FASTKD3**	11	17	502354.78	409722.59	-0.29	FDR > 0.05
**P61224**	**RAP1B**	6	14	26130.10	21213.89	-0.30	FDR > 0.05
**P21695**	**GPD1**	4	16	26841.64	21491.12	-0.32	FDR > 0.05
**P01040**	**CSTA**	8	18	16843.97	13336.92	-0.34	FDR > 0.05
**P02766**	**TTR**	6	16	57930.47	45620.60	-0.34	FDR > 0.05
**O75874**	**IDH1**	5	17	34816.75	26845.59	-0.38	FDR > 0.05
**Q8NG11**	**TSPAN14**	8	17	184687.99	139550.88	-0.40	FDR > 0.05
**Q8N1N4**	**KRT78**	10	18	28554.57	21014.78	-0.44	FDR > 0.05
**P02751**	**FN1**	4	16	27088.45	19794.65	-0.45	FDR > 0.05
**Q6UX06**	**OLFM4**	8	16	128893.41	93717.92	-0.46	FDR > 0.05
**P00918**	**CA2**	5	15	27629.38	18131.95	-0.61	FDR > 0.05
**Q9HCH5**	**SYTL2**	6	15	31420.07	19681.34	-0.67	FDR > 0.05

**P09467**	**FBP1**	10	18	53100.22	32634.18	-0.70	FDR > 0.05
**Q13835**	**PKP1**	5	17	11732.47	6791.69	-0.79	FDR > 0.05
**O60701**	**UGDH**	4	16	25936.42	13951.18	-0.89	FDR > 0.05
**P11498**	**PC**	7	15	60500.74	29689.59	-1.03	FDR > 0.05
**Q9UBC9**	**SPRR3**	4	17	16086.74	6786.75	-1.25	FDR > 0.05
**P78385**	**KRT83**	9	17	23668.96	9211.60	-1.36	FDR > 0.05
**P31327**	**CPS1**	8	14	25076.27	9758.55	-1.36	FDR > 0.05
**P08727**	**KRT19**	11	18	66222.65	23130.54	-1.52	FDR > 0.05
**P04114**	**APOB**	8	17	81382.46	19999.88	-2.02	FDR > 0.05
**P25705**	**ATP5F1A**	6	14	39728.77	9241.56	-2.10	FDR > 0.05
**Q5T750**	**XP32**	5	17	257526.81	58629.80	-2.14	FDR > 0.05
**P16112**	**ACAN**	8	12	192553.48	6572.68	-4.87	FDR > 0.05

Sorted by log2FC COPD vs control, only proteins detected in > 70% of all samples (20/29) are listed

Table S2. Receptors on BALF EVs

ID	protein name	detected in samples control	detected in samples COPD	median intensity control	Median intensity COPD	FC COPD to control	log2FC COPD to control
**Enriched on COPD EVs**							
**Q86X29**	LSR	5/11	14/18	10754.62	29599.78	2.75	1.46
**Q92542**	NCSTN	3/11	13/18	7638.79	17971.40	2.35	1.23
**P13164**	IFITM1;IFITM3;IFITM2	3/11	13/18	14082.71	24930.49	1.77	0.82
**P08575**	PTPRC	3/11	14/18	18893.75	30093.93	1.59	0.67
**P05107**	ITGB2	5/11	16/18	28874.70	43729.40	1.51	0.60
**P11215**	ITGAM	3/11	14/18	11446.00	17328.31	1.51	0.60
**P13726**	F3	5/11	16/18	17478.07	26000.97	1.49	0.57
**Q03135**	CAV1	5/11	14/18	68970.98	88218.31	1.28	0.36
**P26006**	ITGA3	3/11	15/18	16334.63	17816.25	1.09	0.13
**P19397**	CD53	3/11	13/18	52290.19	48478.36	0.93	-0.11
**P11279**	LAMP1	3/11	15/18	31599.47	28388.38	0.90	-0.15
**Present on both COPD and Control EVs**							
**P98088**	MUC5AC	9/11	17/18	7163.80	78424.59	10.95	3.45
**O14493**	CLDN4	9/11	16/18	10389.07	76339.52	7.35	2.88
**P01833**	PIGR	10/11	18/18	25752.37	168829.40	6.56	2.71
**P08962**	CD63	11/11	18/18	15509.93	92003.89	5.93	2.57
**O60603**	TLR2	10/11	14/18	7613.50	36315.76	4.77	2.25
**Q08722**	CD47	8/11	17/18	30198.35	128907.37	4.27	2.09
**P15941**	MUC1	11/11	18/18	114627.77	478975.14	4.18	2.06
**P15311**	EZR	11/11	18/18	30073.13	125195.61	4.16	2.06
**P26038**	MSN	11/11	18/18	38679.39	155342.17	4.02	2.01
**P48509**	CD151	10/11	18/18	18610.87	73690.78	3.96	1.99
**P27487**	DPP4	10/11	16/18	19310.51	71802.37	3.72	1.89
**Q9Y624**	F11R	8/11	15/18	19913.28	71569.84	3.59	1.85
**Q5HYA8**	TMEM67	8/11	13/18	6405.46	22706.98	3.54	1.83
**P21926**	CD9	11/11	18/18	185367.58	612304.22	3.30	1.72
**P27701**	CD82	10/11	18/18	13925.98	45828.48	3.29	1.72
**P16671**	CD36	10/11	17/18	35904.85	105248.47	2.93	1.55
**P55064**	AQP5	8/11	17/18	32881.35	80057.72	2.43	1.28
**P21589**	NT5E	9/11	18/18	15914.30	37610.05	2.36	1.24
**O60635**	TSPAN1	11/11	18/18	81599.34	189403.99	2.32	1.21
**P29972**	AQP1	11/11	18/18	54385.01	119136.54	2.19	1.13
**P60033**	CD81	11/11	18/18	94397.16	126942.65	1.34	0.43
**P16070**	CD44	9/11	16/18	28525.90	32355.83	1.13	0.18
**Q02413**	DSG1	11/11	18/18	15016.69	14262.21	0.95	-0.07
**Q8NG11**	TSPAN14	8/11	17/18	184687.99	139550.88	0.76	-0.40

Proteins are classified as detected if found in >70% of the samples per group

Table S3. Ligands on BALF EVs

Enriched on COPD EVs							
**ID**	protein name	detected in samples control	detected in samples COPD	median control	median COPD	FC COPD to control	log2FC COPD to control
**P80188**	LCN2	5	16	13513.51	91605.94	6.78	2.76
**P02652**	APOA2	7	18	8213.33	49845.71	6.07	2.60
**P01889**	HLA-B	7	15	11863.51	65992.81	5.56	2.48
**P02763**	ORM1	2	15	4261.87	22739.85	5.34	2.42
**P03973**	SLPI	5	17	4688.81	22392.90	4.78	2.26
**P49913**	CAMP	3	16	7343.84	29534.39	4.02	2.01
**P0C0L5**	C4B	6	15	10165.03	39283.80	3.86	1.95
**P31025**	LCN1	4	16	13722.60	45686.12	3.33	1.74
**P02774**	GC	7	16	10640.64	33295.22	3.13	1.65
**P02671**	FGA	6	16	6234.06	19385.57	3.11	1.64
**P18085**	ARF4	6	16	32503.44	92933.91	2.86	1.52
**P19823**	ITIH2	7	16	13629.52	38903.78	2.85	1.51
**P01019**	AGT	3	14	18891.41	51518.06	2.73	1.45
**P02654**	APOC1	5	15	12229.90	33197.90	2.71	1.44
**P02649**	APOE	4	17	22313.33	57849.44	2.59	1.37
**O00592**	PODXL	4	13	9174.47	23671.97	2.58	1.37
**P04004**	VTN	4	15	18657.62	43641.40	2.34	1.23
**P61160**	ACTR2	6	16	17130.95	39225.84	2.29	1.20
**P04196**	HRG	4	16	16816.94	36752.39	2.19	1.13
**P12821**	ACE	6	17	24359.18	50898.48	2.09	1.06
**P43490**	NAMPT	4	14	9497.13	19245.36	2.03	1.02
**O14672**	ADAM10	4	14	24082.33	47027.64	1.95	0.97
**Q9Y490**	TLN1	5	15	7858.96	14113.02	1.80	0.84
**P14174**	MIF	4	15	208177.70	364523.31	1.75	0.81
**O43866**	CD5L	6	16	10115.47	16718.95	1.65	0.72
**P02790**	HPX	4	17	18810.11	30629.91	1.63	0.70
**Q10588**	BST1	2	14	5998.73	9322.37	1.55	0.64
**P01042**	KNG1	5	16	17447.20	27087.80	1.55	0.63
**P02765**	AHSG	5	16	17509.84	27092.54	1.55	0.63
**P13611**	VCAN	2	16	11327.80	17429.38	1.54	0.62
**P06731**	CEACAM5	3	14	36743.91	50241.23	1.37	0.45
**P50552**	VASP	3	16	11167.53	15137.50	1.36	0.44
**P05090**	APOD	4	16	10614.51	12999.29	1.22	0.29
**Q13443**	ADAM9	4	13	15675.53	18833.55	1.20	0.26
**P29350**	PTPN6	3	13	17933.97	21286.27	1.19	0.25
**P01031**	C5	5	16	15572.13	18423.59	1.18	0.24
**P26447**	S100A4	4	16	30173.02	35636.67	1.18	0.24
**Q08554**	DSC1	7	17	11480.60	13469.20	1.17	0.23

**P02746**	C1QB	3	16	19701.71	23025.77	1.17	0.22
**P08571**	CD14	3	14	18100.96	20410.55	1.13	0.17
**Q10589**	BST2	3	15	26911.29	28700.94	1.07	0.09
**P02745**	C1QA	3	15	32851.94	32395.94	0.99	-0.02
**P04003**	C4BPA	2	15	25565.63	24433.46	0.96	-0.07
**P27797**	CALR	5	15	3617.11	3262.29	0.90	-0.15
**Q9UPX8**	SHANK2	4	13	24690.13	22160.82	0.90	-0.16
**P00734**	F2	3	15	13261.53	11359.16	0.86	-0.22
**P25311**	AZGP1	5	16	15592.29	13314.55	0.85	-0.23
**P62330**	ARF6	2	13	45850.06	38333.02	0.84	-0.26
**P11684**	SCGB1A1	4	15	23262.13	17301.17	0.74	-0.43
**P02751**	FN1	4	16	27088.45	19794.65	0.73	-0.45
**P12273**	PIP	7	18	30829.10	22128.42	0.72	-0.48
Present on both COPD and Control EVs							
**P02788**	LTF	9	17	5499.17	140144.44	25.48	4.67
**P02647**	APOA1	11	18	15501.98	328991.03	21.22	4.41
**P05109**	S100A8	9	17	18837.36	320987.78	17.04	4.09
**P02768**	ALB	11	18	73215.32	1072664.00	14.65	3.87
**P01024**	C3	11	18	12868.19	134053.30	10.42	3.38
**P06702**	S100A9	8	16	11734.85	109878.65	9.36	3.23
**P04083**	ANXA1	11	18	49761.63	406865.34	8.18	3.03
**P0C0L4**	C4A	10	16	10357.59	74361.35	7.18	2.84
**P14618**	PKM	10	18	12794.73	80711.59	6.31	2.66
**P02675**	FGB	8	16	8114.62	49456.70	6.09	2.61
**P05155**	SERPING1	8	17	11641.75	67648.52	5.81	2.54
**P01009**	SERPINA1	10	17	18557.37	106177.89	5.72	2.52
**P08174**	CD55	11	18	29733.75	162760.11	5.47	2.45
**P09211**	GSTP1	11	18	27730.71	133975.53	4.83	2.27
**P40199**	CEACAM6	9	17	32876.32	151667.47	4.61	2.21
**P01023**	A2M	9	17	21314.71	95411.09	4.48	2.16
**P04899**	GNAI2	11	18	23756.46	99399.61	4.18	2.06
**P06744**	GPI	9	17	10929.04	41206.30	3.77	1.91
**P07900**	HSP90AA1	8	17	21923.80	81276.92	3.71	1.89
**P13987**	CD59	11	18	109809.55	363903.25	3.31	1.73
**P11142**	HSPA8	11	18	19737.46	64650.77	3.28	1.71
**P05362**	ICAM1	10	18	66720.85	213482.80	3.20	1.68
**P21980**	TGM2	8	18	21517.50	68409.20	3.18	1.67
**P84077**	ARF1	10	18	37530.48	116903.63	3.11	1.64
**Q8IWL1;Q8IWL2**	SFTPA2;SFTPA1	8	17	24691.06	76104.78	3.08	1.62
**P00738**	HP	9	17	32872.28	98180.29	2.99	1.58
**P04439**	HLA-A	9	15	23105.61	66913.24	2.90	1.53
**P0DMV8;P0DMV9**	HSPA1A;HSPA1B	11	18	24940.48	71946.93	2.88	1.53
**P63092;Q5JWF2**	GNAS	9	18	31290.54	87748.36	2.80	1.49
**P35247**	SFTPD	11	16	163502.27	411820.36	2.52	1.33
**P60903**	S100A10	10	16	34386.15	74295.97	2.16	1.11

**P00450**	CP	8	17	19040.43	40592.42	2.13	1.09
**Q01469**	FABP5	11	18	29847.81	60793.57	2.04	1.03
**P02679**	FGG	9	18	19049.89	36785.47	1.93	0.95
**P01008**	SERPINC1	9	17	18293.73	35110.18	1.92	0.94
**P61626**	LYZ	10	18	14816.62	21984.74	1.48	0.57
**P78417**	GSTO1	8	17	31401.39	44087.15	1.40	0.49
**Q96QR1**	SCGB3A1	8	17	21320.32	28022.41	1.31	0.39
**P02787**	TF	9	17	28135.60	36182.37	1.29	0.36
**Q08380**	LGALS3BP	11	18	573287.44	595587.78	1.04	0.06
**P09417**	QDPR	10	18	36741.83	34748.39	0.95	-0.08
**P04114**	APOB	8	17	81382.46	19999.88	0.25	-2.02

Proteins are classified as detected if found in >70% of the samples per group

TABLE S4. List of detected miRNAs in BALF EVs

miRNA	baseMean	mean COPD	mean healthy	log2FoldChange	pvalue	FDR
**hsa-miR-449c-5p**	722.12	1035.01	181.68	2.51	1.31E-06	5.57E-04
**hsa-miR-10a-5p**	146.89	209.86	38.14	2.42	2.48E-06	5.57E-04
**hsa-miR-101-3p**	520.19	401.48	725.24	-0.83	5.21E-06	7.78E-04
**hsa-miR-122-5p**	245.79	370.25	30.81	3.58	9.58E-06	1.07E-03
**hsa-miR-155-5p**	170.26	234.53	59.25	1.89	5.36E-04	1.86E-02
**hsa-miR-191-5p**	1355.72	1575.73	975.69	0.70	7.27E-04	1.92E-02
**hsa-miR-320a-3p**	657.42	804.40	403.55	1.01	6.99E-04	1.92E-02
**hsa-miR-425-5p**	177.11	222.37	98.94	1.17	7.91E-04	1.97E-02
**hsa-miR-128-3p**	661.04	750.25	506.95	0.56	1.04E-03	2.46E-02
**hsa-miR-7-5p**	255.97	308.06	165.98	0.91	1.26E-03	2.82E-02
**hsa-miR-378a-3p**	171.30	209.91	104.61	0.94	2.25E-03	4.58E-02
**hsa-miR-1246**	104.01	143.78	35.30	1.94	2.88E-03	5.60E-02
**hsa-miR-182-5p**	412.13	475.59	302.53	0.67	4.83E-03	7.95E-02
**hsa-miR-361-3p**	126.40	148.51	88.21	0.76	4.48E-03	7.95E-02
**hsa-miR-320c**	130.05	170.58	60.05	1.36	1.29E-02	1.65E-01
**hsa-let-7d-3p**	1233.38	1084.41	1490.70	-0.45	1.68E-02	1.88E-01
**hsa-miR-21-5p**	16788.21	18783.58	13341.65	0.49	2.24E-02	2.07E-01
**hsa-miR-146a-5p**	599.34	705.52	415.95	0.77	2.22E-02	2.07E-01
**hsa-miR-25-3p**	381.08	429.44	297.55	0.53	2.14E-02	2.07E-01
**hsa-miR-30c-5p**	1460.31	1331.12	1683.46	-0.33	2.79E-02	2.20E-01
**hsa-miR-320b**	515.01	652.53	277.48	1.20	2.90E-02	2.23E-01
**hsa-miR-32-5p**	107.08	90.35	135.98	-0.53	3.12E-02	2.26E-01
**hsa-miR-30a-5p**	2145.14	1932.73	2512.05	-0.38	3.70E-02	2.51E-01

**hsa-miR-328-3p**	177.81	199.71	139.98	0.47	5.90E-02	3.22E-01
**hsa-let-7d-5p**	1188.94	1304.93	988.59	0.41	7.57E-02	3.69E-01
**hsa-miR-1307-3p**	437.45	475.15	372.33	0.35	8.20E-02	3.87E-01
**hsa-miR-342-5p**	150.71	139.53	170.03	-0.34	8.46E-02	3.91E-01
**hsa-miR-532-5p**	490.89	435.20	587.07	-0.42	9.60E-02	4.26E-01
**hsa-miR-30e-3p**	581.40	528.39	672.97	-0.32	9.80E-02	4.30E-01
**hsa-miR-200a-3p**	7134.71	7881.98	5843.95	0.43	1.04E-01	4.41E-01
**hsa-miR-183-5p**	669.06	719.24	582.40	0.33	1.14E-01	4.64E-01
**hsa-miR-200b-5p**	275.95	299.53	235.21	0.38	1.13E-01	4.64E-01
**hsa-miR-423-3p**	1395.65	1446.47	1307.87	0.16	1.22E-01	4.67E-01
**hsa-miR-99b-5p**	3208.02	3014.21	3542.77	-0.23	1.29E-01	4.81E-01
**hsa-miR-22-3p**	1600.14	1736.61	1364.43	0.32	1.44E-01	5.16E-01
**hsa-miR-100-5p**	1911.34	2082.40	1615.89	0.36	1.49E-01	5.25E-01
**hsa-miR-148b-3p**	173.68	163.38	191.48	-0.19	1.49E-01	5.25E-01
**hsa-miR-126-3p**	171.22	194.84	130.42	0.55	1.54E-01	5.40E-01
**hsa-miR-200b-3p**	5397.42	5747.96	4791.95	0.26	1.72E-01	5.49E-01
**hsa-miR-423-5p**	4144.54	3860.23	4635.63	-0.26	1.60E-01	5.49E-01
**hsa-miR-1180-3p**	735.64	812.98	602.05	0.44	1.76E-01	5.49E-01
**hsa-miR-143-3p**	625.56	495.62	850.01	-0.78	1.62E-01	5.49E-01
**hsa-miR-2110**	116.75	130.69	92.67	0.54	1.86E-01	5.55E-01
**hsa-miR-26b-5p**	1279.83	1214.07	1393.42	-0.19	2.13E-01	5.82E-01
**hsa-miR-146b-5p**	6209.39	5601.87	7258.74	-0.37	2.47E-01	6.12E-01
**hsa-miR-30e-5p**	414.09	394.83	447.36	-0.18	2.53E-01	6.14E-01
**hsa-miR-744-5p**	212.17	222.47	194.38	0.25	2.54E-01	6.14E-01
**hsa-miR-30a-3p**	904.70	851.42	996.72	-0.23	2.72E-01	6.40E-01

**hsa-miR-28-3p**	225.17	231.65	213.99	0.15	2.91E-01	6.59E-01
**hsa-miR-941**	105.20	113.55	90.78	0.31	2.91E-01	6.59E-01
**hsa-miR-148a-3p**	38236.58	36479.62	41271.34	-0.18	3.17E-01	6.92E-01
**hsa-miR-222-3p**	743.32	707.71	804.83	-0.17	3.45E-01	7.23E-01
**hsa-miR-26a-5p**	42567.89	40999.88	45276.29	-0.14	3.52E-01	7.26E-01
**hsa-let-7a-5p**	23462.72	24791.98	21166.73	0.23	3.52E-01	7.26E-01
**hsa-miR-34b-3p**	524.72	477.00	607.14	-0.34	3.70E-01	7.57E-01
**hsa-miR-200a-5p**	476.43	505.21	426.71	0.24	3.74E-01	7.62E-01
**hsa-miR-27b-3p**	4207.98	4049.56	4481.62	-0.15	3.80E-01	7.66E-01
**hsa-miR-151a-3p**	4163.72	4033.76	4388.20	-0.12	4.08E-01	7.99E-01
**hsa-miR-200c-3p**	13435.14	13813.72	12781.24	0.11	4.25E-01	8.03E-01
**hsa-miR-429**	905.05	944.98	836.09	0.18	4.22E-01	8.03E-01
**hsa-miR-451a**	834.14	944.51	643.51	0.54	4.24E-01	8.03E-01
**hsa-let-7i-5p**	4975.41	5094.12	4770.36	0.09	4.44E-01	8.19E-01
**hsa-miR-3168**	205.26	235.92	152.31	0.57	4.51E-01	8.25E-01
**hsa-miR-375-3p**	6146.53	6443.29	5633.95	0.19	4.62E-01	8.30E-01
**hsa-miR-92b-5p**	474.97	515.19	405.49	0.35	4.65E-01	8.30E-01
**hsa-miR-24-3p**	2227.04	2167.81	2329.35	-0.11	4.97E-01	8.57E-01
**hsa-miR-181b-5p**	187.64	194.99	174.92	0.17	5.01E-01	8.57E-01
**hsa-let-7c-5p**	3397.60	3507.60	3207.61	0.13	5.47E-01	8.66E-01
**hsa-miR-34c-5p**	2425.57	2280.15	2676.75	-0.23	5.45E-01	8.66E-01
**hsa-miR-92a-3p**	1552.98	1596.81	1477.26	0.12	5.44E-01	8.66E-01
**hsa-miR-181a-2-3p**	123.96	115.89	137.89	-0.21	5.45E-01	8.66E-01
**hsa-miR-338-5p**	183.22	170.29	205.57	-0.26	5.66E-01	8.75E-01
**hsa-let-7e-5p**	1488.90	1543.09	1395.29	0.15	5.99E-01	8.80E-01

**hsa-miR-221-3p**	145.73	155.00	129.73	0.16	5.93E-01	8.80E-01
**hsa-miR-98-5p**	513.09	498.82	537.73	-0.10	6.27E-01	8.97E-01
**hsa-miR-186-5p**	375.90	381.79	365.75	0.09	6.28E-01	8.97E-01
**hsa-miR-30d-5p**	65790.39	63235.00	70204.25	-0.15	6.58E-01	9.07E-01
**hsa-miR-140-3p**	105.27	107.63	101.19	-0.18	6.77E-01	9.08E-01
**hsa-miR-203a-3p**	2149.73	2226.77	2016.65	0.14	7.08E-01	9.14E-01
**hsa-miR-224-5p**	453.84	466.94	431.21	0.11	7.02E-01	9.14E-01
**hsa-miR-340-5p**	109.76	112.07	105.77	0.10	7.37E-01	9.22E-01
**hsa-miR-99a-5p**	18168.19	17883.21	18660.43	-0.06	7.49E-01	9.25E-01
**hsa-miR-141-3p**	802.19	810.93	787.09	0.06	7.63E-01	9.29E-01
**hsa-miR-30b-5p**	373.56	363.33	391.25	-0.11	7.82E-01	9.29E-01
**hsa-miR-103a-3p**	190.10	191.25	188.12	0.08	7.81E-01	9.29E-01
**hsa-let-7f-5p**	11449.13	11582.06	11219.53	0.05	8.29E-01	9.52E-01
**hsa-let-7b-5p**	20308.44	20509.03	19961.97	0.04	8.37E-01	9.54E-01
**hsa-miR-125a-5p**	3972.86	3929.26	4048.18	-0.04	8.60E-01	9.58E-01
**hsa-miR-181a-5p**	1176.04	1166.14	1193.14	-0.02	8.94E-01	9.65E-01
**hsa-miR-125b-5p**	653.90	657.56	647.59	0.02	8.97E-01	9.65E-01
**hsa-miR-184**	459.54	455.95	465.73	-0.07	9.01E-01	9.65E-01
**hsa-miR-16-2-3p**	154.56	153.72	156.00	0.03	9.07E-01	9.65E-01
**hsa-miR-27a-3p**	3411.82	3406.01	3421.87	-0.01	9.34E-01	9.78E-01
**hsa-miR-92b-3p**	2656.15	2673.45	2626.26	0.03	9.48E-01	9.80E-01
**hsa-miR-29a-3p**	1247.23	1263.52	1219.10	0.01	9.55E-01	9.80E-01
**hsa-let-7g-5p**	2143.57	2147.56	2136.66	0.01	9.70E-01	9.81E-01

List of 96 miRNAs detected in BALF EVs with a basemean of >100.
